# LncRNA‐Mediated TPI1 and PKM2 Promote Self‐Renewal and Chemoresistance in GBM

**DOI:** 10.1002/advs.202402600

**Published:** 2024-09-28

**Authors:** Changxiao Yang, Ziwei Li, Kaifu Tian, Xiangqi Meng, Xinyu Wang, Dan Song, Xuan Wang, Tianye Xu, Penggang Sun, Junzhe Zhong, Yu Song, Wenbin Ma, Yuxiang Liu, Daohan Yu, Ruofei Shen, Chuanlu Jiang, Jinquan Cai

**Affiliations:** ^1^ Department of Neurosurgery The Second Affiliated Hospital of Harbin Medical University Harbin 150086 China; ^2^ Future Medical Laboratory The Second Affiliated Hospital of Harbin Medical University Harbin 150086 China; ^3^ Beijing Tiantan Hospital Capital Medical University Beijing 100070 China; ^4^ Department of Neurosurgery Union Hospital Tongji Medical College Huazhong University of Science and Technology Wuhan Hubei 430074 China; ^5^ The Sixth Affiliated Hospital of Harbin Medical University Harbin 150086 China

**Keywords:** GBM, Linc00942, PKM2, TPI1, TMZ resistance

## Abstract

Temozolomide (TMZ) resistance is one of the major reasons for poor prognosis in patients with glioblastoma (GBM). Long noncoding RNAs (lncRNAs) are involved in multiple biological processes, including TMZ resistance. Linc00942 is a potential regulator of TMZ sensitivity in GBM cells is shown previously. However, the underlying mechanism of TMZ resistance induced by Linc00942 is unknown. In this study, the sequence of Linc00942 by rapid amplification of cDNA ends assay in TMZ‐resistant GBM cells is identified and confirmed that Linc00942 contributes to self‐renewal and TMZ resistance in GBM cells. Chromatin isolation by RNA purification followed by mass spectrometry (ChIRP‐MS) and followed by Western blotting (ChIRP‐WB) assays shows that Linc00492 interacted with TPI1 and PKM2, subsequently promoting their phosphorylation, dimerization, and nuclear translocation. The interaction of Linc00942 with TPI1 and PKM2 leads to increased acetylation of H3K4 and activation of the STAT3/P300 axis, resulting in the marked transcriptional activation of SOX9. Moreover, the knockdown of SOX9 reversed TMZ resistance induced by Linc00492 both in vitro and in vivo. In summary, Linc00942 strongly promotes SOX9 expression by interacting with TPI1 and PKM2 is found, thereby driving self‐renewal and TMZ resistance in GBM cells. These findings suggest potential combined therapeutic strategies to overcome TMZ resistance in patients with GBM.

## Graphical abstract

TMZ resistance is one of the major reasons for the poor prognosis of patients with GBM. Linc00942, the sequence of which is identified, interacts with TPI1 and PKM2, subsequently promoting their phosphorylation, dimerization, and nuclear translocation. The interaction leads to the elevated transcriptional activation of SOX9 and contributes to self‐renewal and TMZ resistance, providing potential strategies to overcome therapeutic resistance.

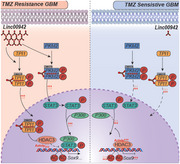

## 1 Introduction

Glioblastoma (GBM) is the most common primary cancer of the central nervous system, and although patients with GBM receive various treatments, their prognosis remains poor.^[^ ^1^ ^]^ Owing to the extreme heterogeneity and complex mechanisms of tumor immune escape, GBM is highly resistant to existing therapies, including temozolomide (TMZ).^[^ ^2^, ^3^ ^]^ In recent years, aberrant transcriptional activation of long noncoding RNAs (lncRNAs) has been considered a key factor underlying TMZ resistance in GBM.^[^ ^4^ ^]^ Our previous studies have shown that lncRNAs could affect sensitivity to TMZ treatment in many ways; for example, GBM cell‐derived lnc‐TALC mediates M2 polarization of microglia, and extrinsic lnc‐TALC promotes C5a release in M2 microglia by activating the p38/MAPK pathway, which contributes to DNA repair and TMZ resistance in GBM cells.^[^ ^5^ ^]^ Recent studies have demonstrated that lncRNAs interact with metabolic enzymes and contribute to cancer development.^[^ ^6^ ^]^ However, whether the binding of lncRNAs to metabolic enzymes alters therapeutic resistance in GBM remains largely unknown.

According to recent research, metabolic enzymes are involved in transcriptional regulation in many ways, particularly histone modification. Histone acetylation requires essential molecular groups supplied by metabolic substrates.^[^ ^7^ ^]^ In addition, certain metabolic substrates alter the activity of histone acetylases and deacetylases.^[^ ^8^, ^9^ ^]^ Specific metabolic enzymes are transported to the nucleus and mediate their corresponding metabolic substrates, thereby regulating histone acetylation. Recent studies have revealed that TPI1, a metabolic enzyme involved in glycolysis, can be phosphorylated by mTOR signaling and transported into the nucleus. Furthermore, higher TPI1 expression results in a lower concentration of DHAP, thereby inhibiting the promotion of histone deacetylases by DHAP.^[^ ^10^ ^]^ Moreover, metabolism enzymes can directly interact with various proteins and transcription factors, modulate related pathways, and activate or inhibit target genes.^[^ ^11^, ^12^ ^]^ PKM2 is one of the most important metabolic enzymes and is highly expressed in multiple cancers.^[^ ^13^, ^14^, ^15^ ^]^ Previous studies have shown that PKM2 interacts with STAT3 and increase the activity of STAT3 signaling by promoting the phosphorylation of STAT3.^[^ ^16^, ^17^, ^18^ ^]^ Therefore, whether the interactions between metabolic enzymes and lncRNAs can regulate biological processes in GBM requires further investigation.

SOX9 is a high‐mobility group box (HMG‐box) factor that is a key regulator of stem cell population maintenance.^[^ ^19^, ^20^ ^]^ Recent studies have shown that SOX9 expression is crucial for stemness maintenance in various types of cancers and that SOX9 knockdown significantly blocks cancer stem cell phenotype development.^[^ ^21^, ^22^, ^23^ ^]^ SOX9 is highly expressed in GBM and is considered an essential factor driving GBM stem cell phenotype and self‐renewal, which significantly remodels sensitivity to chemotherapy.^[^ ^24^, ^25^, ^26^ ^]^

According to our previous study, Linc00942 is highly expressed in multiple TMZ‐resistant GBM cell lines and exhibits potential regulatory capacity in the context of TMZ resistance. However, the mechanisms underlying Linc00942‐mediated TMZ resistance remain unclear. In the current study, we confirmed that Linc00942 is a novel regulator of TMZ sensitivity in GBM and that knockout of Linc00942 inhibits self‐renewal and TMZ resistance in TMZ‐resistant GBM cells. Linc00942 activates SOX9 expression by interacting with both TPI1 and PKM2, thereby driving self‐renewal and TMZ resistance in GBM cells. Our results suggest a new strategy for overcoming TMZ resistance in GBM.

## 2 Results

### 2.1 Linc00942 Promotes TMZ Resistance and STAT3 Signaling Activity in GBM Cells

**Figure 1:**
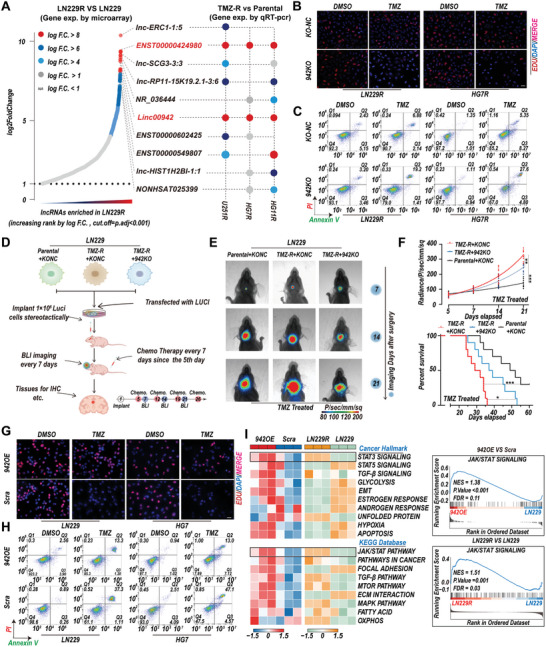
Linc00942 promotes TMZ resistance and STAT3 signaling activation in GBM cells. A) Microarray and qRT‐PCR analysis of the top 10 elevated lncRNAs in LN229R and parental cells. B) EdU assay of LN229R and HG7R cells transfected with KONC or 942KO and treated with TMZ (100 µM). Scale bar = 50 µm. C) Apoptosis rate detected by flow cytometry assay of LN229R and HG7R cells transfected with KONC or 942KO and treated with TMZ (200 µM). D) Diagram of orthotopic mouse model construction and further experiments. E) Bioluminescence images of mice bearing tumors derived from LN229R cells transfected with KONC or 942KO and corresponding parental LN229 cells transfected with KONC (*n* = 10). F) Quantification of bioluminescence curves (up) and Kaplan‒Meier survival curves (down) of mice bearing the indicated GBM cells (*n* = 10 mice). G) EdU assay of LN229 and HG7 cells transfected with Linc00492 or scrambled with TMZ (100 µM) Scale bar = 50 µm. H) Apoptosis rate detected by flow cytometry assay of LN229 and HG7 cells transfected with Linc00492 or scramble with TMZ (200 µM). I) ssGSEA and GSEA based on the transcriptome data. The top differential gene sets in Linc00492‐overexpressing LN229 are indicated. *p*‐values in (F) were determined by log‐rank Test (bottom) and two‐way ANOVA followed by Tukey's multiple comparison test (top). Significant results are presented as *p < 0.05; **p < 0.01; or ***p < 0.001.

To determine the differential lncRNA expression profiles between TMZ‐resistant GBM cells and parental cells, we analyzed lncRNA microarrays^[^ ^27^ ^]^ and found that Linc00942 was highly expressed in a variety of TMZ‐resistant GBM cells (**Figure** **1**A) and had a potential biological function in promoting TMZ resistance in gliomas according to siRNA screening assays (Figure S1, Supporting Information). Subsequently, we identified the sequence of Linc00942 by rapid amplification of cDNA ends (RACE) assay, which showed that Linc00942 consisted of 2 exons with a full length of 2230 nt, transcribed on chromosome 12 from site 1500521 to site 1504440 (5′‐3′) (Figure S2A–D, Supporting Information). Open reading frame (ORF) (Figure S2E,F, Supporting Information) and coding ability analyses (Figure S2G,H, Supporting Information) revealed that Linc00942 lacked coding ability. Linc00942 was enriched in both the nucleus and cytoplasm, as detected by fluorescence in situ hybridization (FISH) and quantitative real‐time polymerase chain reaction (qRT‒PCR) (Figure S2I–K, Supporting Information). To further explore the effect of Linc00942 on TMZ resistance in GBM, we knocked out Linc00942 in TMZ‐resistant LN229R, U251R, HG7R, and HG11R cells using CRISPR/Cas9 (Figure S3A, Supporting Information) and confirmed the effect of Linc00942 on TMZ sensitivity both in vitro and in vivo. In in vitro experiments, Linc00942 knockout resulted in a lower IC50 of TMZ and a lower EdU‐positive rate (Figure 1B; Figure S3B–D, Supporting Information). The apoptotic rates of Linc00942 knockout TMZ‐resistant LN229R, U251R, HG7R, and HG11R cells increased under TMZ treatment compared to control cells (Figure 1C; Figure S3E–G, Supporting Information). These results suggest that knockout of Linc00942 led to significantly reduced cell viability and DNA replication under TMZ treatment. Glioma stemness is one of the main factors influencing drug resistance, and TMZ‐resistant glioma cells have a greater capacity for self‐renewal after TMZ treatment.^[^ ^28^, ^29^ ^]^ In this study, we performed a tumorsphere formation assay, extreme limiting dilution analysis (ELDA), and flow cytometry (Figure S3H–M, Supporting Information). Results showed that knockout of Linc00942 significantly suppressed the self‐renewal of TMZ‐resistant LN229R, U251R, HG7R, and HG11R cells. In vivo, we established orthotopic mouse models using TMZ‐resistant LN229R GBM cells, with or without Linc00942 knockout and paired them with parental LN229 GBM cells (Figure 1D). Bioluminescence imaging showed that Linc00942 knockout attenuated the difference in the therapeutic effect of TMZ between the LN229R and parental groups (Figure 1E). Mice bearing LN229R cells exhibited significantly poorer survival rates than those bearing parental LN229 cells. In addition, the knockout of Linc00942 prolonged the survival time of mice bearing LN229R cells (Figure 1F). These results indicated that Linc00942 contributes to TMZ resistance in GBM cells and that knockout of Linc00942 partly restored TMZ sensitivity in TMZ‐resistant GBM cells. Next, we established a Linc00942‐overexpressing GBM model (Figure S4A, Supporting Information) to verify whether overexpression of Linc00942 promotes stemness and TMZ resistance in GBM cells. Compared to scrambled GBM cells, the overexpression of Linc00942 in LN229, U251, HG7, and HG11 cells significantly improved cell viability, DNA replication, and self‐renewal during TMZ treatment (Figure 1G,H; Figure S4B–J, Supporting Information). To further investigate how Linc00492 promotes TMZ resistance in GBM cells, we performed RNA‐seq on Linc00492‐overexpressing LN229 cells and scrambled LN229 cells to identify transcriptomic changes. A volcano plot showed that 716 genes were significantly upregulated in Linc00492‐overexpressing LN229 cells (Figure S5A, Supporting Information). We also confirmed the differentially expressed mRNAs in LN229R cells using a gene‐ranking plot based on the microarray data published in our previous study^[^ ^27^ ^]^ (Figure S5B, Supporting Information). The Venn diagram showed that 278 genes were co‐upregulated in both Linc00492‐overexpressing LN229 cells and TMZ‐resistant LN229R GBM cells (Figure S5C, Supporting Information). Transcription factor enrichment analysis using UCSC showed that these co‐upregulated genes were significantly enriched in STAT targets (Figure S5D, Supporting Information). ssGSEA and GSEA suggested that STAT3 signaling and the JAK/STAT pathway, but not STAT5 signaling, were activated in LINC00492‐overexpressing LN229 and TMZ‐resistant LN229R GBM cells (Figure 1I). Collectively, these data demonstrate that Linc00492 significantly increased TMZ resistance in GBM cells and was related to activated STAT3 signaling.

### 2.2 TPI1 and PKM2 are Direct Binding Partners and Downstream Factors Regulated by Linc00942

**Figure 2:**
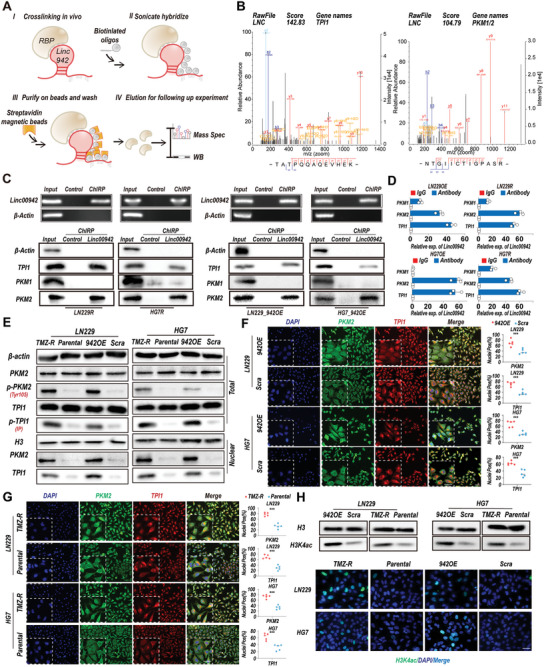
TPI1 and PKM2 are functional binding partners of Linc00942. A) Schematic of the ChIRP‐MS assays to detect Linc00492 binding factors. B) High‐resolution MS/MS spectra of Linc00492 oligos and TPI1/PKM2 cross‐linked peptide pairs. C) ChIRP‐WB analysis of Linc00942 binding proteins in the indicated cell lines. An agarose assay was used for quality control. D) RIP‐qPCR experiments using the indicated antibodies and specific primers to detect Linc00942 (*n* = 3). E. WB analysis of Linc00942 binding factors TPI1 and PKM2. The total expression, phosphorylation, and nuclear expression levels of both proteins were detected. Pan‐phospho antibody was used for detecting phosphorylation of TPI1 by IP. F, G) IF and colocalization analysis of PKM2 (green) and TPI1 (red) in the indicated cells. Colocation analysis was performed by ImageJ, scale bar = 50 µm (*n* = 5). H. WB and IF assays of H3K4ac in the indicated cells, scale bar = 50 µm. Values in (D) represent the mean ± SD from 3 independent experiments. Values in (G and F) represent the mean ± SD from 5 independent experiments. p‐values (F and G) were determined using a two‐tailed Student's t‐test. Significant results are presented as **p < 0.01 or ***p < 0.001.

lncRNAs often exert their diverse biological functions by interacting with RNA‐binding proteins.^[^ ^5^, ^30^ ^]^ To further elucidate the potential mechanism of Linc00942, we used ChIRP, followed by mass spectrometry (ChIRP‐MS), to identify potential Linc00942‐binding factors in GBM cells (**Figure** **2**A; Figure S6A–D, Supporting Information). Nineteen specific biotin‐labeled Linc00942 probes were used to capture Linc00942 from the total cellular extracts of TMZ‐resistant GBM cells LN229R (Table S1, Supporting Information), whereas negative probes were used as a control. Twelve proteins were significantly enriched with Linc00942 probes versus negative probes (Figure S6E, Supporting Information). Among these proteins, we found that TPI1 and PKM1/2, 2 glycolytic enzymes, were the top 2 Linc00942 binding factors (Figure 2B). We further confirmed that TPI1 and PKM2, but not PKM1, interacted significantly with Linc00942 using ChIRP‐WB (Figure 2C; Figure S6F, Supporting Information) and RNA immunoprecipitation (RIP) assays (Figure 2D; Figure S6G,H, Supporting Information) in both TMZ‐resistant and Linc00942‐overexpressing GBM cells. Recent studies have shown that the phosphorylation and nuclear translocation of TPI1 and PKM2 result in significant changes in biological processes, leading to therapy resistance and oncogene expression in many cancers.^[^ ^31^, ^32^ ^]^ Therefore, we measured the expression levels of TPI1 and PKM2 by WB and found no significant differences in the total expression levels of TPI1 and PKM2. However, phosphorylation and nuclear translocation of TPI1 and PKM2 were significantly increased in TMZ‐resistant GBM cells and Linc00942‐overexpressing GBM cells (Figure 2E; Figure S7A, Supporting Information). We also confirmed the significant nuclear translocation of TPI1 and PKM2 in TMZ‐resistant GBM cells and Linc00942‐overexpressing GBM cells by immunofluorescence (IF) assay and colocalization analysis (Figure 2F,G). In addition, various metabolic enzymes and substrates have been reported as regulators of histone modifications. Both TPI1 and PKM2 promote histone acetylation through nuclear translocation, especially nuclear translocation of TPI1, which leads to significant histone acetylation in several types of cancer cells by modulating histone deacetylase (HDAC) activity.^[^ ^7^, ^10^ ^]^ Therefore, we performed HDAC activity assays and WB to determine whether changes in histone acetylation occurred in TMZ‐resistant GBM cells and Linc00492‐overexpressing GBM cells. Finally, we found that the activity of HDAC3 was significantly decreased and the acetylation of H3K4 was increased (Figure 2H; Figure S7B,C, Supporting Information). Taken together, these results revealed that Linc00942 could bind to TPI1 and PKM2 and regulate histone acetylation via the activity of HDAC3 in GBM cells.

### 2.3 Interactions of TPI1 and PKM2 with Linc00942 are Essential for Linc00942‐Mediated TMZ Resistance

**Figure 3:**
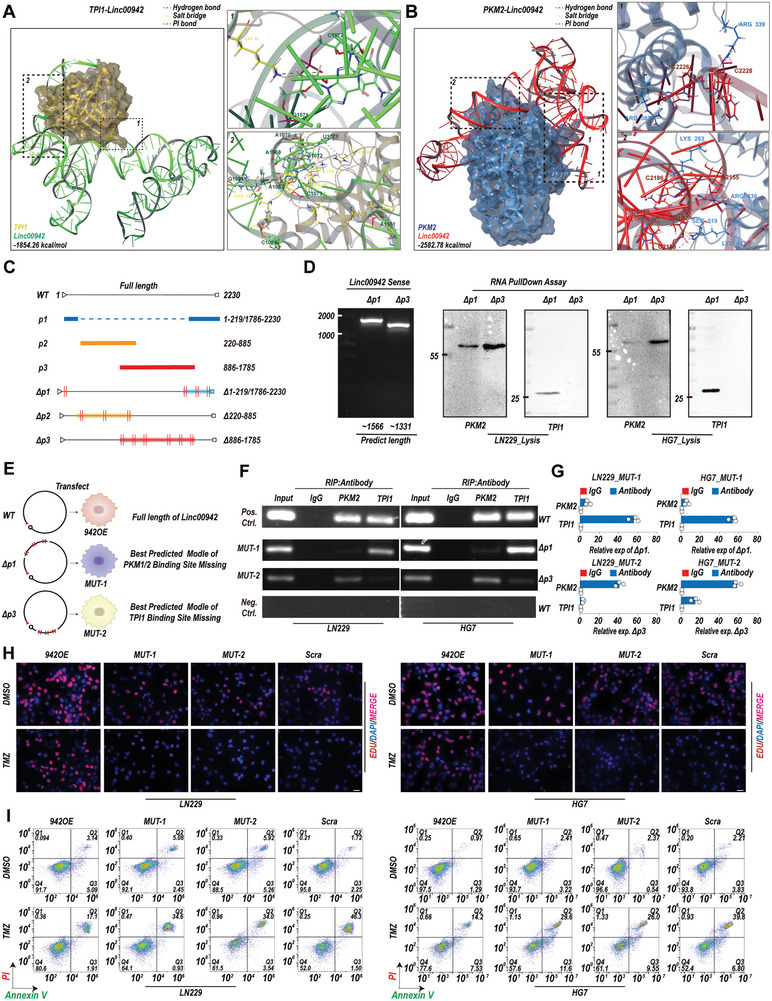
Linc00942‐mediated TMZ resistance depends on its interaction with TPI1 and PKM2. A, B) Diagram showing how Linc00942 interacts with TPI1 and PKM2. Interaction force, binding interface, interactions at the amino acid level, electrostatic forces, and predicted binding energies are shown. C) Diagram of the substructure and mutant sequence of Linc00942. D) WB analysis of TPI1 and PKM2 following biotin‐labeled mutant Linc00942 (ΔP1 and ΔP3) pull‐down assays in the indicated cells. An agarose assay was used as quality control. E) Diagram of the construction of ΔP1‐ and ΔP3‐overexpressing GBM cells, named MUT‐1 and MUT‐2, respectively. F, G) RIP assays followed by PCR and qPCR (*n* = 3) were performed on the indicated cells using specific TPI1 or PKM2 antibodies and specific primers to detect Linc00942. H) EdU assay of LN229 and HG7 cells treated with TMZ (100 µM). The corresponding scrambled cells were used as controls. Scale bar = 50 µm. I) Apoptosis rate detected by flow cytometry in LN229 and HG7 cells treated with TMZ (200 µM). The corresponding scrambled cells were used as controls. Values in G) represent the mean ± SD from 3 independent experiments.

Both RNA and proteins fold into complex 3D structures to perform a broad range of cellular functions.^[^ ^33^, ^34^ ^]^ To further investigate the mechanism by which Linc00942 binds to TPI1 and PKM2, we performed a molecular docking analysis to identify the specific binding site. Alpha fold was used to predict the 3D structures of TPI1 and PKM2^[^ ^35^ ^]^ (Figure S8A,B, Supporting Information). The 3D RNA server was used to establish a 3D model of Linc000942 based on the minimal folding free energy (MFE) structure,^[^ ^36^ ^]^ and the best model was selected for further analysis (Figure S8C,D, Supporting Information). A molecular docking model was constructed using HDOCK to predict the optimal docking site.^[^ ^37^ ^]^ Schrödinger software was used to evaluate the quality of molecular docking and to calculate the interaction force, details of the binding interface, interactions at the amino acid level, electrostatic forces, and predicted binding energies. The results of molecular docking analysis showed that the interaction between Linc00942/TPI1 (−1854.26 kcal mol^−1^) and Linc00942/PKM2 (−2585.78 kcal mol^−1^) is highly stable. Linc00942 interacted with TPI1 through a kissing loop at 960–1147/1547–1620 nt of Linc00942 through salt bridges, hydrogen bonds, and Pi‐cationic bonds. The first‐rank binding site of PKM2 was located at 2100–2230/1–10 nt of Linc00942 through a salt bridge and hydrogen bond (**Figure** **3**A,B; Figure S8E,F, Supporting Information). Several studies have shown that lncRNAs interact with and modulate their corresponding proteins through specific substructures.^[^ ^38^, ^39^ ^]^ MFE and 3D structural analyses revealed that Linc00942 is composed of 3 main substructures. These 3 substructures were named p1 (1–219/1786–2230 nt), p2 (220–885 nt), and p3 (886‐1785 nt). To verify the binding sites of TPI1 and PKM2, in combination with the results of molecular docking analysis, we constructed mutant Linc00942 ΔP1 (del1‐219/1786‐2230 nt) and ΔP3 (del886–1785 nt) plasmids according to the predicted binding site of Linc00942 and TPI1/PKM2 (Figure 3C). A biotin‐labeled RNA pull‐down assay showed that TPI1 interacted with ΔP1 (del1‐219/1786–2230 nt), and PKM2 mainly bound to ΔP3 (del886–1785 nt) (Figure 3D). The parental GBM cells and corresponding TMZ‐resistant Linc00942 knockout cells transfected with ΔP1 and ΔP3 were designated MUT‐1 and MUT‐2 GBM cells, respectively (Figure 3E; Figure S9A,B, Supporting Information). We further performed RIP assays in MUT‐1 and MUT‐2 GBM cells, and the results showed that TPI1 mainly bound to mutant Linc00942 in MUT‐1 cells, and PKM2 mainly bound to mutant Linc00942 in MUT‐2 (Figure 3F,G; Figure S9C,D, Supporting Information). We then investigated the role of the interaction of Linc00942 with TPI1 and PKM2 in TMZ resistance. The IC50 assay showed that both mutations of Linc00942 partly attenuated the TMZ resistance induced by Linc00942 overexpression in GBM cells. However, both MUT‐1 and MUT‐2 GBM cells exhibited higher TMZ resistance than scrambled GBM cells (Figure S9E, Supporting Information). EdU assays indicated that Linc00942 mutations led to a significantly decreased proportion of EdU‐positive cells (Figure 3H; Figure S9F, Supporting Information) and increased apoptotic rates (Figure 3I; Figure S9G, Supporting Information) compared with that among Linc00942‐overexpressing GBM cells during TMZ treatment. In addition, mutations in Linc00942 weakened its ability of Linc00942 to promote tumorsphere formation (Figure S9H–J, Supporting Information). These results indicate that Linc00942‐induced TMZ resistance depends on interactions with both TPI1 and PKM2.

### 2.4 Linc00942 Promotes the Acetylation of H3K4 and Activation of STAT3 Through the Interactions of TPI1 and PKM2

**Figure 4:**
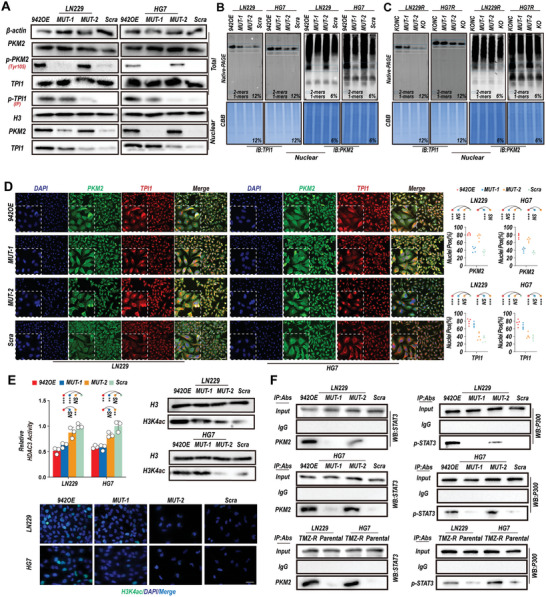
Linc00942 triggers phosphorylation, dimerization, and nuclear translocation of TPI1 and PKM2, resulting in H3K4 acetylation and STAT3/p300 activation. A) WB analysis of Linc00942 binding factors TPI1 and PKM2 in the indicated cells. The total expression, phosphorylation, and nuclear translocation levels of both proteins were detected. A pan‐phospho antibody was used to detect the phosphorylation of TPI1 by IP. B, C) Native PAGE analysis of TPI1 and PKM2 polymers in the indicated cells. D) IF and colocalization analysis of PKM2 (green) and TPI1 (red) in the indicated cells. Colocation analysis was performed by ImageJ, scale bar = 50 µm (*n* = 5). E. HDAC3 assay (*n* = 3), WB and IF of acetylation modification in indicated cells, Scale bar = 50 µm. F. IP assay of the interaction between PKM2/STAT3 and STAT3/p300 in the indicated cell lines. Values in (D) represent the mean ± SD from 5 independent experiments. Values in E) represent the mean ± SD from 3 independent experiments. P‐values in (D and E) were determined using a two‐tailed Student's t‐test. Significant results are presented as **p < 0.01 or ***p < 0.001.

Based on these results, we concluded that TMZ resistance induced by Linc00942 is dependent on the interaction between TPI1 and PKM2. However, whether TPI1 and PKM2 are regulated by direct interaction remains unclear. Therefore, we measured the expression of TPI1 and PKM2 in MUT‐1 and MUT‐2 GBM cells. We found that the phosphorylation and nuclear expression levels of TPI1, but not PKM2, were significantly higher in MUT‐1 GBM cells than in scrambled GBM cells. In addition, phosphorylation and nuclear translocation of PKM2 were significantly increased in MUT‐2 GBM cells, but not in MUT‐1 GBM cells (**Figure** **4**A; Figure S10A, Supporting Information). The quaternary structure of proteins is closely related to their phosphorylation; therefore, phosphorylation can lead to significant changes in their biological functions. Both TPI1 and PKM2 can undergo complicated polymerizations associated with crucial biological processes.^[^ ^17^, ^40^ ^]^ Therefore, we further analyzed the polymerization of TPI1 and PKM2 by native PAGE in nuclear extraction using native PAGE. The results showed that the expression of the TPI1 dimer was significantly increased in Linc00942‐overexpressing and MUT‐1 GBM cells. In addition, expression of the dimer, but not the tetramer, of PKM2 was significantly increased in Linc00942‐overexpressing and MUT‐2 GBM cells (Figure 4B; Figure S10B, Supporting Information). Furthermore, TPI1 dimer and PKM2 dimer were highly expressed in TMZ‐resistant LN229R, U251R, HG7R, and HG11R cells, which could be inhibited by mutation or knocking out of Linc00942. (Figure 4C; Figure S10C, Supporting Information). IF assays and colocalization analysis revealed that the nuclear translocation of TPI1 was increased in MUT‐1 GBM cells and that the nuclear translocation of PKM2 was increased in MUT‐2 GBM cells (Figure 4D). These results indicate that mutations in the TPI1/PKM2 binding sites of Linc00942 regulated the complicated polymerization processes of TPI1 and PKM2. Next, we determined HDAC3 activity and H3K4ac expression in MUT‐1 and MUT‐2 GBM cells. Compared to scrambled cells, MUT‐1 GBM cells showed a significant reduction in HDAC3 activity and higher H3K4ac expression, and the results were similar for Linc00942‐overexpressing GBM cells (Figure 4E; Figure S10D, Supporting Information). Recent studies have shown that PKM2 is a direct regulator of STAT3 and that the nuclear translocation of PKM2 significantly promotes the phosphorylation of STAT3.^[^ ^16^, ^17^ ^]^ Therefore, we performed immunoprecipitation, followed by WB to determine whether Linc00942 promotes the interaction between PKM2 and STAT3. The results showed that the interaction between PKM2 and STAT3 was significantly enhanced in Linc00942‐overexpressing GBM cells and MUT‐2 GBM cells compared to that in scrambled cells. Moreover, a similar increase in the interaction between PKM2 and STAT3 was found in TMZ‐resistant LN229R, U251R, HG7R, and HG11R cells compared to that in the parental cells. In addition, we confirmed a markedly activated STAT3/P300 axis in Linc00942‐overexpressing GBM cells, TMZ‐resistant GBM cells, and MUT‐2 GBM cells (but not in MUT‐1 cells), suggesting that the interaction between Linc00942 and PKM2 plays a key role in activating the STAT3 pathway (Figure 4F; Figure S10E, Supporting Information). Taken together, our results demonstrate that the post‐translational modifications of TPI1 and PKM2 regulated by interactions with Linc00492 contribute to the acetylation of H3K4 and activation of the STAT3 signaling pathway in GBM cells.

### 2.5 Linc00942 Promotes the Transcriptional Activation of SOX9, Regulating TMZ Resistance in GBM Cells

**Figure 5:**
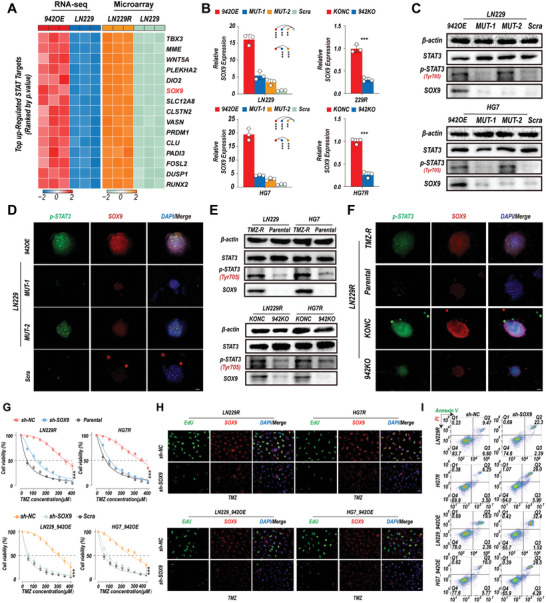
Linc00942 induces marked upregulation of SOX9, driving self‐renewal and TMZ resistance in GBM. A) Differential analysis of the top 15 co‐upregulated STAT target genes in Linc00942‐overexpressing LN229 cells and LN229R cells. B) q‐PCR analysis of SOX9 in the indicated cells with specific primers (*n* = 3). C–F) WB and sphere‐IF assay of STAT3, p‐STAT3, and SOX9 in the indicated cells. Scale bar = 50 µm. G. IC50 analysis of TMZ in Linc00942‐overexpressing or LN229R and HG7R cells transfected with sh‐NC or sh‐SOX9, corresponding to scramble or parental cells used as controls (*n* = 3). H. EdU assay of LN229 and HG7 cells transfected with sh‐NC or sh‐SOX9 with TMZ (100 µM), corresponding scramble or parental cells used for control, Scale bar = 50 µM. I. Apoptosis rate detected by flow cytometry assay of LN229 and HG7 cells transfected with sh‐NC or sh‐SOX9 with TMZ (200 µM). Values in B and G) represent the mean ± SD from 3 independent experiments. P‐values in (B) were determined by a two‐tailed Student's t‐test. P‐values in (G) were determined by two‐way ANOVA followed by Tukey's multiple comparison test. Significant results are presented as **p < 0.01 or ***p < 0.001.

H3K4 acetylation in specific promoter regions can be regulated by both HDAC3 and p300, which can contribute to marked transcriptional changes.^[^ ^41^, ^42^ ^]^ To determine how Linc00942‐induced transcriptional alterations contribute to TMZ resistance in GBM cells, we analyzed the expression of STAT target genes using a heat map (**Figure** **5**A). The results showed that SOX9 was significantly upregulated in Linc00942‐overexpressing LN229 cells and TMZ‐resistant LN229R GBM cells. Recent studies have reported that SOX9 is a key regulator of stemness in GBM, and high SOX9 expression results in drug resistance in various cancers, including TMZ resistance in GBM cells.^[^ ^24^, ^25^, ^26^, ^43^ ^]^ We further detected the expression of SOX9 by qRT‐PCR. The results showed that the expression level of SOX9 was significantly increased in Linc00942‐overexpressing GBM cells and TMZ‐resistant GBM cells compared to that in scrambled/parental GBM cells. Moreover, knockout of Linc00942 inhibited SOX9 expression in TMZ‐resistant LN229R, U251R, HG7R, and HG11R cells. However, only a slight increase in SOX9 expression was observed in MUT‐1 and MUT‐2 GBM cells (Figure 5B; Figure S11A–C, Supporting Information). WB and tumor sphere IF assays confirmed the qRT‐PCR results. In addition, p‐STAT3 expression was significantly increased in Linc00942‐overexpressing GBM cells, MUT‐2 GBM cells, and TMZ‐resistant GBM cells compared to scrambled/parental GBM cells, which is consistent with our previous results (Figure 5C–F; Figure S11D,E, Supporting Information). To further elucidate the mechanisms of SOX9 transcription, we downloaded ChIP‐Seq data for p300 (GSM935545), STAT3 (GSM935591), HDAC3 (GSE127356), and H3K4ac (GSM521894) from the Gene Expression Omnibus (GEO) database (Figure S12A, Supporting Information). p300, STAT3, HDAC3, and H3K4ac were significantly enriched in the SOX9 promoter region (Figure S12B, Supporting Information). We then performed a ChIP‐qPCR assay on LN229 and HG7 cells. The results showed that HDAC3 enrichment in the SOX9 promoter region was significantly decreased in Linc00942‐overexpressing and MUT‐1 GBM cells and that the interaction of STAT3 with the SOX9 promoter region was significantly increased in Linc00942‐overexpressing and MUT‐2 GBM cells compared with scrambled control cells. However, STAT3 enrichment in the SOX9 promoter region was much lower in MUT‐2 GBM cells than in Linc00942‐overexpressing cells. Dramatically increased levels of p300 and H3K4ac in the SOX9 promoter region were detected only in Linc00942‐overexpressing GBM cells (Figure S12C, Supporting Information). Changes in the levels of p300, STAT3, HDAC3, and H3K4ac were also confirmed in TMZ‐resistant LN229R, U251R, HG7R, and HG11R cells, and these effects were reversed by knockout of Linc00942 (Figure S12D,E, Supporting Information). Next, we knocked down SOX9 in Linc00942‐overexpressing LN229, U251, HG7, and HG11 cells and TMZ‐resistant LN229R, U251R, HG7R, and HG11R cells using shRNA (Figure S13A, Supporting Information). The IC50 assay showed that the reduction in SOX9 expression significantly inhibited the viability of Linc00942‐overexpressing GBM cells and TMZ‐resistant GBM cells during TMZ treatment (Figure 5G; Figure S13B, Supporting Information). IF and flow cytometry assays showed that knockdown of SOX9 resulted in a significantly decreased EdU‐positive rate (Figure 5H; Figure S13C,D, Supporting Information) and increased apoptotic rate (Figure 5I; Figure S13E–G, Supporting Information) in Linc00942‐overexpressing GBM cells and TMZ‐resistant GBM cells. Sphere formation, ELDA, and flow cytometry assays showed that the knockdown of SOX9 significantly suppressed the self‐renewal of Linc00942‐overexpressing GBM cells and TMZ‐resistant GBM cells (Figure S13H–K, Supporting Information). These results showed that Linc00492 promoted TMZ resistance in GBM in a manner dependent on the marked transcriptional activation of SOX9 through the suppression of HDAC3 activity and STAT3 activation.

### 2.6 In Vivo Experiments Validated that Linc00942 Promotes TMZ Resistance by Interacting with TPI1 and PKM2

**Figure 6:**
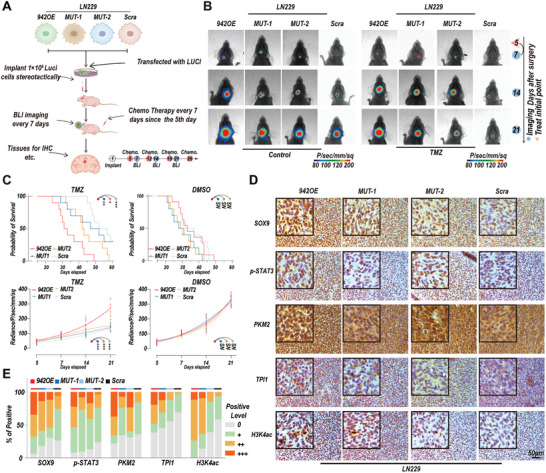
Linc00942 promotes TMZ resistance in vivo. A) Schematic of the construction and further experiments of orthotopic mouse models with the indicated cells. B) Bioluminescence images of mice bearing tumors derived from Linc00492‐overexpressing, MUT‐1, MUT‐2, and scramble LN229 cells treated with DMSO or TMZ. C) Quantification of Kaplan‒Meier survival curves (up) and bioluminescence curves (down) of mice bearing Linc00492‐overexpressing, MUT‐1, MUT‐2, and scramble LN229 cells. (*n* = 10 mice). D, E) IHC staining of SOX9, TPI1, PKM2, and H3K4AC in consecutive brain sections of orthotopic GBM mice treated with Linc00492 overexpressing, MUT‐1, MUT‐2, or scrambled LN229 cells. The histogram represents a quantitative evaluation of the IHC assay. Scale bar = 50 µm. P‐values in (C) were determined using the Log‐rank Test (top) and 2‐way ANOVA, followed by Tukey's multiple comparison test (bottom). Significant results are presented as *p < 0.05, **p < 0.01, and ***p < 0.001.

To further verify that Linc00942 regulates TMZ resistance via interaction with TPI1 and PKM2, we established orthotopic mouse models bearing GBM xenografts by transplantation of Linc00942‐overexpressing, MUT‐1, MUT‐2, and scrambled LN229 cells (**Figure** **6**A). Five days after GBM cell implantation, the mice were treated intraperitoneally with TMZ or DMSO (60 mg kg^−1 ^per mouse) every 7 days. Simultaneously, the mice underwent bioluminescence tomography every 7 d, starting on day 7. Bioluminescence imaging showed that the efficacy of TMZ treatment was significantly inhibited in mice with GBM derived from Linc00942‐overexpressing cells. Moreover, mutations in Linc00942 (MUT‐1 and MUT‐2) in LN229 cells partially inhibited TMZ resistance in vivo (Figure 6B). Survival and bioluminescence analyses indicated that TMZ treatment significantly prolonged the survival time of mice bearing MUT‐1, MUT‐2, and scrambled LN229 cells compared to that of those bearing Linc00942‐overexpressing GBM cells (Figure 6C). Immunohistochemistry (IHC) results showed that the nuclear translocation of TPI1 and expression of H3K4ac were significantly increased in mice bearing Linc00942‐overexpressing and MUT‐1 LN229 cells. Nuclear translocation of PKM2 and expression of p‐STAT3 significantly increased in mice bearing Linc00942‐overexpressing and MUT‐2 LN229 cells. A marked increase in the expression of SOX9 was observed only in mice bearing Linc00942‐overexpressing LN229 cells (Figure 6D,E). In addition, we performed an IHC assay in mice bearing GBM cells derived from LN229 and TMZ‐resistant LN229R cells, with or without Linc00942 knockout. The results showed that SOX9, p‐STAT3, and H3K4ac are highly expressed in TMZ‐resistant LN229R cells. Furthermore, TPI1 and PKM2 translocation was significantly increased in TMZ‐resistant LN229R mice. Knockout of Linc00942 attenuated these effects (Figure S14A,B, Supporting Information). Additionally, IHC results showed that the expression of Ki‐67 was significantly decreased and the TUNEL expression level was enhanced in mice bearing Linc00942 knockout cells under TMZ treatment (Figure S14C,D, Supporting Information). Furthermore, we established orthotopic models using Linc00942‐overexpressing LN229 cells and TMZ‐resistant LN229R GBM cells with or without SOX9 knockdown. Survival and bioluminescence analyses indicated that SOX9 knockdown significantly restored TMZ sensitivity in vivo (Figure S14E,F, Supporting Information). In addition, we performed a FISH assay combined with multiplex immunohistochemistry on TMZ‐treated recurrent GBM and primary GBM samples from patients. The results showed that the expression of Linc00942 was increased in TMZ‐treated recurrent GBM samples. Moreover, the expression level of SOX9 and nuclear translocation of TPI1 and PKM2 were higher in recurrent TMZ‐treated GBM tissues than in newly diagnosed GBM tissues (Figure S15, Supporting Information). Overall, our results revealed that the TMZ resistance induced by Linc00942 is related to a marked increase in SOX9 expression, which was regulated by the direct interaction of TPI1 and PKM2 with Linc00942.

## 3 Discussion

With advances in technology, various novel treatments have emerged; however, most therapeutics fail to improve the prognosis of patients with GBM.^[^ ^2^ ^]^ TMZ remains the first‐line chemotherapeutic drug for glioma; unfortunately, the high heterogeneity of GBM drives rapid resistance to TMZ treatment.^[^ ^44^ ^]^ Multiple factors appear to play a role in treatment resistance, including an immunosuppressive tumor environment, maintenance of tumor stemness, and activation of the DNA damage repair pathway.^[^ ^45^ ^]^ Multiple lncRNAs have been reported as regulators of TMZ sensitivity.^[^ ^46^ ^]^ However, the biological functions of only a small proportion of these lncRNAs have been elucidated.

Based on our previous study, we confirmed that lnc‐TALC and Linc00942 were highly expressed in TMZ‐resistant GBM cells. Our previous study showed that histone acetylation can increase O^6^‐methylguanine‐DNA methyltransferase (MGMT) expression in TMZ‐resistant GBM cells. MGMT, which encodes the DNA repair protein O6‐alkylguanine DNA alkyltransferase, is ubiquitously expressed in human tissues and is often upregulated in malignant tissues.^[^ ^47^ ^]^ MGMT promoter methylation correlates with decreased progression‐free and overall survival in patients with GBM treated with alkylating agents. Our previous work and that of other teams found that functional dysregulation of DNA damage repair contributes to chemoresistance in GBM, including mismatch repair.^[^ ^48^, ^49^ ^]^ However, the mechanism of TMZ resistance induced by Linc00942 remains unknown. Linc00942 has been reported to be a tumor‐promoting factor in several cancers^[^ ^50^, ^51^ ^]^; however, the sequence of Linc00942 in previous studies was based on predictions. In this study, we identified the sequence of Linc00942 in GBM cells using RACE. FISH and qRT‒PCR demonstrated that Linc00942 was expressed in both the nucleus and cytoplasm, which was crucial for guiding further experiments. The nuclear lncRNA NEAT1 promotes oncogenic transcription by interacting with EZH2.^[^ ^52^ ^]^ Further experiments verified Linc00942‐mediated TMZ resistance both in vitro and in vivo. We analyzed the microarray data of TMZ‐resistant LN229R GBM cells and parental LN229 cells and performed RNA‐seq and gene set enrichment analysis in Linc00942‐overexpressing LN229 cells and scramble LN229 cells. These results verified that the STAT3 signaling pathway, which has multiple functions in various cancers,^[^ ^53^ ^]^ is involved in Linc00942‐induced TMZ resistance.

lncRNAs are involved in diverse biological processes that mostly depend on their interactions with RNA‐binding proteins (RBPs).^[^ ^54^ ^]^ lncRNAs serve as scaffolds for distinct proteins that promote specific molecular complex formation.^[^ ^55^ ^]^ For example, the lincRNA HOTAIR serves as a central platform for the interaction between PRC2 and LSD1, resulting in H3K27 and H3K4 methylation.^[^ ^56^ ^]^ In addition, the interaction between lncRNAs and RBPs directly regulates the modification and stability of the corresponding proteins. A previous study reported that GAS5 directly interacts with YAP, thereby promoting its phosphorylation and subsequent ubiquitin‐mediated degradation of YAP in colorectal cancer.^[^ ^57^ ^]^ In this study, we performed ChIRP‐MS and ChIRP‐WB to determine the partner proteins of Linc00942 and found that TPI1 and PKM2, 2 key metabolic enzymes, were the 2 main binding proteins of Linc00942.

Tumor cells regulate the classical functions of metabolic enzymes to support rapid proliferation and also regulate a variety of complex cellular activities and tumor progression through nonclassical/nonmetabolic functions.^[^ ^11^, ^58^ ^]^ Recent research has revealed that lncRNAs can facilitate metabolic enzyme complex assembly to form a higher‐efficiency substrate channel, driving metabolic reprogramming via classical pathways.^[^ ^6^, ^59^ ^]^ However, whether the interactions between lncRNAs and metabolic enzymes contribute to the nonclassical/nonmetabolic functions of metabolic enzymes remains unclear. TPI1 and especially PKM2 have been considered chemotherapy resistance‐promoting factors; PKM2 has been reported as a STAT3 activator.^[^ ^16^, ^18^ ^]^ Therefore, we wondered whether interactions with Linc00942 induced functional changes in TPI1 and PKM2. Further experiments showed that the phosphorylation and nuclear translocation of both proteins were promoted in Linc00942‐overexpressing GBM cells and TMZ‐resistant cells. Previous studies have shown that both TPI1 and PKM2 regulate the acetylation of histones, thereby contributing to tumor progression.^[^ ^7^, ^10^ ^]^ Our results showed a significant decrease in HDAC3 activity and a significant increase in H3K4ac expression in Linc00942‐overexpressing LN229, U251, HG7, and HG11 cells and in TMZ‐resistant LN229R, U251R, HG7R, and HG11R cells.

lncRNAs interact with proteins via specific substructures, and the disruption of specific structures leads to the loss of the ability of these lncRNAs to bind to the corresponding proteins.^[^ ^6^, ^39^ ^]^ To clarify the mechanism of the interaction of Linc00942 with TPI1 and PKM2, we performed a molecular docking analysis to identify the distinct binding sites of TPI1 and PKM2 at Linc00942. In this case, we constructed 2 mutant Linc00942 vectors, ΔP1 (predicted PKM2 binding sites missing) and ΔP3 (predicted TPI1 binding sites missing); we confirmed that TPI1 interacts with ΔP1 and that PKM2 mainly interacts with ΔP3. GBM cells transfected with ΔP1 and ΔP3 (MUT‐1 and MUT‐2) lost the TMZ resistance and self‐renewal phenotypes induced by Linc00942. Further experiments validated that GBM cells with mutations in the TPI1 and PKM2 binding sites affected the expression levels, polymer formation ability, phosphorylation levels, and nuclear translocation of both proteins. Protein modification, polymer formation, and subcellular localization play important roles in the biological function of specific proteins.^[^ ^60^ ^]^ These results indicate that Linc00942 promotes the phosphorylation, dimerization, and nuclear translocation of TPI1 and PKM2 through direct interactions. Recent studies have reported that the nuclear translocation of TPI1 promotes histone acetylation by suppressing HDAC activity.^[^ ^10^ ^]^ Consistently, our results revealed that the reduction in HDAC3 activity related to the interaction of Linc00942 with TPI1 resulted in the elevation of H3K4ac. In addition, we verified that the interaction of Linc00942 with PKM2 activates the Stat3/p300 axis and increases p‐STAT3 expression.

Glioma stem cells (GSCs) are the core cause of tumor growth, angiogenesis, and therapeutic resistance in GBM.^[^ ^61^ ^]^ According to transcriptome data, SOX9, a GSC driving factor, was strongly upregulated. SOX9 has been broadly studied in the field of cancer stem cells, and high SOX9 expression is related to the promotion of self‐renewal.^[^ ^19^, ^24^, ^43^ ^]^ Recent studies have revealed that SOX9 contributes to chemotherapy resistance and is associated with DDR.^[^ ^62^, ^63^ ^]^ However, marked transcriptional activation was observed in Linc00492‐overexpressing LN229, U251, HG7, and HG11 cells and TMZ‐resistant LN229R, U251R, HG7R, and HG11R cells, but not in MUT‐1 and MUT‐2 cells, and knockout of Linc00942 significantly suppressed SOX9 expression. SOX9 regulation by HDAC3 and STAT3 has been reported in several studies, and the inhibition of HDAC3 and activation of STAT3 leads to the elevation of SOX9 expression.^[^ ^64^, ^65^ ^]^ Based on the ChIP‐Seq data, the ChIP‒qPCR assay revealed that the enrichment of H3K4ac and p300 at the SOX9 promoter was significantly increased in Linc00942‐overexpressing GBM cells and TMZ‐resistant GBM cells, but not in MUT‐1 and MUT‐2 cells. Further experiments confirmed that SOX9 knockdown markedly restored TMZ sensitivity and inhibited cell self‐renewal. We constructed an orthotopic mouse model to verify the in vivo results. Limited efficiency of TMZ treatment and marked SOX9 elevation were observed in Linc00942‐overexpressing GBM cell‐bearing mice, but not in MUT‐1 and MUT‐2 cell‐bearing mice. We also verified that knockout of Linc00942 inhibited SOX9 expression in TMZ‐resistant cells in vivo and that knockdown of SOX9 significantly prolonged the survival of mice bearing Linc00492‐overexpressing GBM cells and TMZ‐resistant GBM cells.

**Figure 7:**
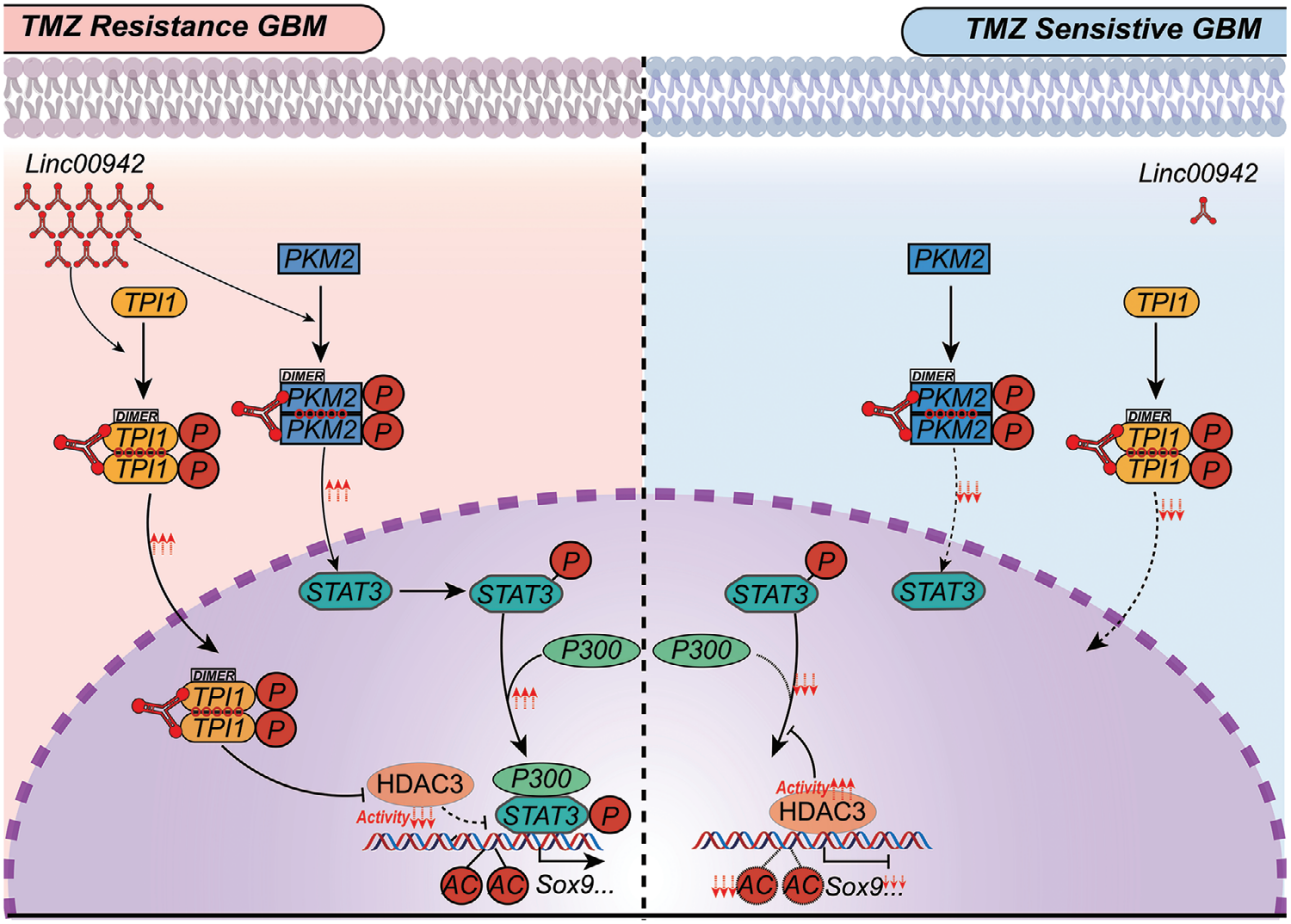
Diagram summarizing findings from the current study. Left, high Linc00942 expression triggers phosphorylation, dimerization, and nuclear translocation of TPI1 and PKM2 via direct interaction. Subsequently, acetylation of the SOX9 promoter at H3K4 is promoted by the modulation of HDAC3 activity and the Stat3/p300 axis, thereby promoting SOX9 expression and TMZ resistance. Right, phosphorylation, dimerization, and nuclear translocation of TPI1 and PKM2 are suppressed in GBM cells with low Linc00942 expression. High HDAC3 activity and low Stat3/p300 axis activation result in lower SOX9 expression.

In conclusion, our study revealed that Linc00942, a novel lncRNA, interacts with TPI1 and PKM2, thereby strongly promoting SOX9‐dependent self‐renewal and TMZ resistance in GBM cells. Our findings enhance the understanding of TMZ resistance mediated by the interactions between lncRNAs and metabolic enzymes through nonmetabolic functions, which may provide a novel therapeutic strategy (**Figure** **7**).

## 4 Conclusion

In summary, our study revealed that lncRNAs are involved in the nonmetabolic functions of metabolic enzymes. Further experiments proved that Linc00942 is a lncRNA that promotes TMZ resistance. Linc00942‐mediated TMZ resistance depends on the transcriptional activation of SOX9, which is triggered by the interaction of Linc00942 with TPI1 and PKM2.

## 5 Experimental Section

### Cell Lines and Cell Culture

The human GBM cell lines LN229 and U251 were purchased from the Chinese Academy of Sciences Cell Bank. Patient‐derived GBM cells HG7 and HG11 were isolated from discarded GBM specimens according to the previous studies.^[^ ^27^, ^66^ ^]^ In brief, tumor tissue was washed, mechanically minced in MACS C Tubes (MiltenyiBiotec, Germany), and digested with 0.1% trypsin (Invitrogen, USA),10 U mL^−1^ of DNase I (Promega, USA) at 37 °C for 45 min and neutralized by minimum essential medium (MEM‐α) containing 10% fetal bovine serum (FBS). Red blood cells were lysed using the ACK lysis buffer (Beyotime, Shanghai, China). The washed tissues were passed through a 100 µm cell strainer and cultured in Dulbecco's Modified Eagle Medium (DMEM/F12) supplemented with N2, B27 (Gibco, USA), 20 ng mL^−1^ human fibroblast growth factor‐basic (bFGF, Sino Biological, China). All the cell lines were authenticated using the STR assay (Genetic Testing Biotechnology).

### Drug Treatment

TMZ (Selleck, USA) was dissolved in DMSO and stored at −80 °C for further experiments. TMZ was added to the glioma culture medium (CM) at a concentration of 100 µM or the indicated concentration for 72 h or an indicated time in vitro and in vivo (60 mg kg^−1^ via intraperitoneal injection).

### Establishment of TMZ‐Resistant Cells

The process of establishing TMZ‐resistant LN229, U251, and HG7 cells was consistent with that described in the previous study.^[^ ^27^ ^]^ In addition, HG11 cells into 96‐well plates at 3000 cells per well, and the half‐maximal inhibitory concentration (IC50) of TMZ was evaluated using the CCK‐8 (Dojindo, Japan) assay were seeded. The cells were treated with TMZ at an IC50 1/50 concentration in six‐well plates. The TMZ dose was increased until the HG11 cells grew stably and each dose of TMZ was maintained for 15 days until the end of the 5th month. GBM cells with induced TMZ resistance were named 229R, U251R, HG7R, and HG11R.

### CCK‐8 Assay

Cell Counting Kit 8 (Dojindo, Japan) was used to calculate the viability of GBM cells in 96‐well plates. CCK‐8 solution (10 µL) was added to each well and incubated at 37 °C for 1 h. Then, the specific counts at OD 450 nm were detected using the BioTek Gen5 system (BioTek, USA).

### EdU Assay

For the EdU assay, the cells subjected to different treatments were seeded onto tissue culture‐treated slides (Nest, Rahway, NJ, USA) for 24 h before incubation with EdU (Ribobio, Guangzhou, China). The cells were incubated in a complete medium with 10 µM EdU for 24 h and then fixed and labeled with Apollo 567/488 (RiboBio, Guangzhou, China) according to the manufacturer's protocol. DAPI was used for nucleic acid staining, and images were captured using a fluorescence microscope.

### Tumorsphere Formation Assay and ELDA

For the sphere formation assay, GBM cells were digested and cultured in fresh serum‐free medium (SFM, DMEM/F12 1:1 medium containing 20 ng mL^−1^ fibroblast growth factor, 20 ng mL^−1^ epidermal growth factor, 0.50 × B27, and N2) in ultralow attachment microplates (Corning Costar, Lowell, MA, USA) and allowed to form tumor spheres. Fresh SFM was added every 3 days for 14 days.

For ELDA, spherical cells were dissociated into single‐cell suspensions and seeded into 96‐well plates such that the density in each well ranged from 10 to 100 cells, with 10 replicates for each gradient. The number of tumor spheres (diameter ≥ 50 µm) in each well was determined after 14 days of incubation, and the sphere formation efficiency was calculated by Extreme Limiting Dilution Analysis (http://bioinf.wehi.edu.au/software/elda)

### Flow Cytometry

Flow cytometry was used for apoptosis analysis and the determination of cell surface markers. For cell apoptosis analysis, Annexin V‐FITC Apoptosis Detection Kit (BD Biosciences) was used according to the manufacturer's instructions. The apoptosis rate was measured by flow cytometry (FC) using a BD FACSCanto II and analyzed using FlowJo. FITC Anti‐Human CD44 antibody (Elabscience, China) and APC Anti‐Human CD117/c‐Kit a‐antibody (Elabscience, China) were used to detect cell surface markers. Briefly, cells were stained in phosphate‐buffered saline (PBS) containing 2% bovine serum albumin (BSA) (BioFroxx, China) at 4 °C for 1 h with antibodies as indicated. The expression of cell surface markers was determined by flow cytometry (FC) using a BD Accuri C6 cytometer and analyzed using FlowJo. Details of the antibodies used in the flow cytometry assays are provided in Table S2 (Supporting Information).

### RNA Isolation, PCR, and qRT‐PCR

TRIzol reagent (Invitrogen, USA) was used for total RNA extraction according to the manufacturer's instructions. The PrimeScript RT Reagent Kit (TaKaRa, Japan) was used to synthesize cDNAs following the manufacturer's instructions. A SYBR Green kit (TaKaRa, Japan) was used for qRT‐PCR. Each group consisted of at least 3 replicates. Both conventional PCR and qRT‐PCR were performed using a CFX96 Touch Real‐Time PCR Detection System (Bio‐Rad, USA), and 1% agarose gel electrophoresis (AGE) was used to detect the PCR products. The indicated genes were normalized to β‐actin. The qRT‐PCR data were analyzed by the 2^−△△Ct^ method. PCR primers were designed using a primer design tool (http://www.ncbi.nlm.nih.gov/tools/primerblast/). Primer sequences used in this study are listed in Table S3 (Supporting Information).

### High‐Throughput Sequencing

The transcriptome data for LN229R and LN229 cells were obtained using an Agilent custom human lncRNA and mRNA microarray (SHBIO Biotechnology Corporation, Shanghai, China). The raw data were normalized by the R package limma using the quantile algorithm to normalize the raw count. Gene ranking plots representing differentially regulated genes were generated using the R package limma. The microarray data were published in the previous study and deposited in the NCBI GEO database (www.ncbi.nlm.nih.gov/geo) under accession numbers GSE113510 and GSE131781.

RNA expression profiling of LN229_942OE and LN229_Scra cells was performed by RNA sequencing according to a previous study, with 3 replicates for each group. Briefly, total RNA was extracted using TRIzol reagent (Invitrogen, USA). The NEBNext Ultra RNA Library Prep Kit for Illumina (NEB, USA) was used for the construction of sequencing libraries following the manufacturer's recommendations. An Agilent 2100 bioanalyzer was used for the quality control of total RNA and sequencing libraries. Library preparations were sequenced on an Illumina HiSeq 2500 platform, and 125 bp paired‐end reads were generated. Clean reads were annotated using HISAT2 to identify their locations in the reference genome. DEseq2 was used for the differential expression analysis between the LN229_942OE and LN229_Scra groups. Volcano plots and heat maps showing the differentially expressed genes were generated using ggplot2 and pheatmap.

### RNA Pull‐Down

Mutated Linc00942 RNA ΔP1 (del 1–219/1786‐2230 nt) and ΔP3 (del 886–1785 nt) were transcribed with a Ribo RNAmax‐T7 Biotin Labeled RNA Synthesis Kit (Ribobio, Guangzhou, China) in vitro. Biotin‐labeled RNA was heated and incubated to form secondary and tertiary structures and mixed with whole‐cell extracts in RIP buffer for 1 h. Biotin‐labeled RNA was then pulled down using streptavidin magnetic beads (MCE, USA). RBPs were then detected by western blot (WB) assay.

### Rapid Amplification of cDNA Ends (RACE)

Total RNA was isolated using the TRIzol reagent (TIANGEN, China) according to the manufacturer's instructions. 5′ RACE and 3′ RACE analyses were performed with 1 µg of total RNA. The GoScript Reverse Transcription System (Promega, USA) was used according to the manufacturer's instructions. The RACE PCR products were separated on a 1% agarose gel. Gene‐specific primers used for PCR are listed in Table S4 (Supporting Information).

### Coding Potential Analysis

For coding potential analysis, 4 different methods, including ORF Finder from NCBI, phyloCSF, coding probability from the coding potential assessment tool (CPAT), and coding potential score from the coding potential calculator, were used to calculate the coding potential of Linc00942. Putative proteins encoded by Linc00942 were performed using ORF Finder. NEAT1 served as a control noncoding gene. Glyceraldehyde‐3‐phosphate dehydrogenase (GAPDH) and F‐actin (ACTF) were used as controls. PhyloCSF as phyloCSF  =  0 and a coding probability of  36.4% as the threshold was defined.^[^ ^27^ ^]^

### Cell Transfection

LN229, U251, HG7, and HG11 cells were infected with Linc00942 overexpression lentivirus to generate LN229_942OE, U251_942OE, HG7_942OE, and HG11_942OE cells, respectively. To generate mutated Linc00942‐overexpressing cells, LN229, U251, HG7 and HG11 cells were transfected with plasmids carrying ΔP1 (del 1–219/1786–2230 nt) and ΔP3 (del 886–1785 nt). Linc00942 knockout was performed using a CRISPR‐Cas9‐based system following the manufacturer's instructions as previously described.^[^ ^67^ ^]^ In brief, cells were transfected by the viruses containing the CAS9 gene in an infection duration of 48 h. Then, the cells were selected by fresh culture medium containing 3 mg mL^−1^ puromycin for 7 days. Subsequently, virus‐carrying small guide (sg) RNAs designed for Linc00942 were used to infect the cells for 24 h. CRISPR/ Cas9/Linc00942‐sgRNA plasmid pairs and lentiviruses were synthetized and purchased from Genechem Company (Shanghai, China).

Short hairpin RNA‐expressing plasmids were used for SOX9 knockdown. Linc00942 overexpression lentivirus and corresponding gene expression plasmid were synthesized by GeneChem (Shanghai, China). All plasmids were transfected using Lipo2000 (Thermo Fisher, USA) following the manufacturer's instructions. siRNA was used for the RNAi assay and transfected using Lipo2000, and the detailed methods were published in the previous study.^[^ ^27^ ^]^ Details of the sgRNA sequences for the Linc00942 knockout are shown in Table S5 (Supporting Information). shRNA and siRNA sequences are listed in Table S6 (Supporting Information). Detailed information regarding the vectors used in this study is provided in Table S7 (Supporting Information).

### Western Blot

For SDS‐PAGE, the total protein was extracted using a prechilled RIPA buffer (Solarbio, China) combined with 1% proteinase and phosphatase inhibitor cocktails (Selleck, China). After measurement by a NanoDrop 2000C spectrophotometer (NanoDrop Products) in compliance with the manufacturer's instructions, all protein samples were subjected to 7.5%/10%/12.5% sodium dodecyl sulfate‐polyacrylamide gel (EpiZyme Scientific) electrophoresis, and the PVDF membranes blocked in a 5% milk‐TBST solution were incubated overnight at 4 °C with primary antibodies (Table S2, Supporting Information). All membranes were incubated with HRP‐labeled mouse immunoglobulin G (IgG) secondary antibodies (Zsbio Store‐bio, China) and HRP‐labeled rabbit IgG secondary antibodies (Zsbio Store‐bio, China). A chemiluminescence reagent kit (Boster) was used to visualize the protein bands.

Native PAGE was used to detect polymerization. For native PAGE, the total protein was extracted using a prechilled native lysis buffer (Solarbio, China) combined with 1% proteinase and phosphatase inhibitor cocktails (Selleck, China). All native protein samples were loaded with native gel sample loading buffer (Beyotime, China) without boiling. Electrophoresis was performed at 4 °C.

### Immunofluorescence (IF)

Cells were transferred to 24‐well plates and plated on cell coverslips (WHB‐24‐CS, China) for 24 h. Cells were fixed with 4% formaldehyde in PBS for 20 min at room temperature. The cells were washed thrice in chilled PBS for 5 min each, treated with 0.3% Triton‐X100 (Thermo Fisher Scientific, USA), and blocked in blocking buffer (5% bovine serum albumin diluted in warm PBS; BioFroxx, China) for 60 min at room temperature. Primary antibodies (Table S2, Supporting Information) diluted in 5% BSA in PBS were incubated at 4 °C overnight. After washing thrice with PBS, the cells were incubated with FITC‐labeled anti‐IgG antibodies (Alexa Fluor 488 and 594, Thermo Fisher) for 1 h at room temperature. DAPI (Sigma, USA) was used to stain cell nuclei. Subcellular protein localization was visualized using a fluorescence microscope (Nikon C2, Japan).

### Fluorescence In Situ Hybridization (FISH)

RNA ISH probes were purchased from GeneCam Corporation (GeneCam, China), and fluorescence in situ hybridization was performed using an RNA in situ hybridization kit (BersinBio, China) according to the manufacturer's instructions. Briefly, the indicated TMZ‐resistant cells were transferred onto coverslips overnight. The cells were fixed with 4% formaldehyde for 10 min and treated with 0.3% Triton‐X100 diluted in PBS for 10 min. Next, the coverslips were treated with 10% proteinase K at 37 °C for 10 min. Then, the cells were incubated in hybridization mixtures at 37 °C for 30 min for hybridization. Then, the cells were incubated with Linc00942p‐FISH Probe Mix at 37 °C overnight. After incubation, the cells were washed with 4 × saline sodium citrate (SSC, containing 0.1% Tween‐20) at 42 °C for 5 min, followed by 2 × SCC at 42 °C for 5 min and 1 × SCC at 42 °C for 5 min. DAPI (Sigma, USA) was used to counterstain the nuclei, and high‐resolution images were obtained using a fluorescence microscope (Nikon C2, Japan). The probe sequences are listed in Table S8 (Supporting Information).

### Chromatin Isolation by RNA Purification (ChIRP)

The cells were resuspended in precooled PBS and crosslinked with 3% formaldehyde at room temperature on an end‐to‐end shaker for 30 min. The reaction was quenched by the addition of glycine (125 mM) for 5 min. The solution was spun at 1000 RCF for 3 min and the supernatant was discarded. One milliliter of lysis buffer was added to 2 × 10^7^ cells and the cell lysate was sonicated in an ice‐water bath until it was no longer turbid. After spinning at top speed, the supernatant was transferred to 2 volumes of hybridization buffer and mixed well to incubate at 37 °C. Subsequently, a prebound probe (4 for TT, 1 for NC and PC, and 100 pmol per 2 × 10^7^ cells) was added to the streptavidin beads for 30 min. After mixing with cell lysate, the beads were hybridized at 37 °C overnight on an end‐to‐end shaker. The beads were then washed 5 times with 1 mL prewarming wash buffer for 5 min. After the final wash, 1/20 of the beads were transferred for qPCR analysis. One hundred microliters of elution buffer and 20 U of benzonase were used to elute protein at 37 °C for 1 h. The supernatant was transferred to a new low‐binding Eppendorf tube to elute the beads again with 100 µL of elution buffer. After combining the 2 supernatants, the crosslink at 95 °C for 30 min was reversed. The protein was precipitated with 0.1% SDC and 10% TCA at 4 °C for 2 h. After spinning at the top speed, the pellets were washed 3 times with precooled 80% acetone. The probe sequences for Linc00942 are listed in Table S9 (Supporting Information).

### Liquid Chromatography‐Tandem Mass Spectrometry (LC‐MS/MS) Analysis

Half of the peptides in each sample were separated and analyzed using a nano‐UPLC (EASY‐nLC1200) coupled to a Q‐Exactive mass spectrometer (Thermo Finnigan). A reversed‐phase column (100 µm, ID × 15 cm, Reprosil‐Pur 120 C_18_‐AQ, 1.9 µm) was used to perform the separation. H_2_O with 0.1% FA and 2% ACN (phase A), and 80% ACN and 0.1% FA (phase B) formed the mobile phases. A 120 min gradient at a 300 nL min^−1^ flow rate to separate the sample was executed: Gradient B: 8%–30% for 92 min, 30%–40% for 20 min, 40%–100% for 2 min, 100% for 2 min, 100 to 2% for 2 min and 2% for 2 min. The Orbitrap analyzer was used for data‐dependent acquisition in profile and positive mode at a resolution of 7 × 10^4^ (200 m z^−1^) and an m/z range of 350–1600 for MS1, and the resolution was set to 1.75 × 10^4^ with a dynamic first mass for MS2. The automatic gain control target for MS1 was set to 1 × 10^6^ with a maximum IT of 100 ms and 5 × 10^4^ for MS2 with a maximum IT of 200 ms. The top 10 most intense ions were fragmented by higher‐energy collisional dissociation with a normalized collision energy of 27% and an isolation window of 2 m z^−1^. The dynamic exclusion time window was set at 30 s.

### Molecular Docking Analysis

The structures of TPI1 and PKM2 were downloaded from the AlphaFold Database (https://www.alphafold.ebi.ac.uk). The MFE structure of Linc00942 was predicted using RNA‐fold analysis (http://rna.tbi.univie.ac.at/). A 3D model of Linc00942 was predicted by 3D‐RNA (https://biophy.hust.edu.cn/new/3dRNA). The molecular interaction model of Linc00942‐TPI1 and Linc00942‐PKM2 were constructed using the HDOCK Server (http://hdock.phys.hust.edu.cn/). To evaluate the binding quality of both models, Schrödinger software was used to predict the specific binding energy and calculate the electrostatic forces of the amino acid‐level interactions at the binding interface. Briefly, the binding interfaces of Linc00942 in the 2 models were predicted by 3DRNA and then processed using the Nucleotide Preparation Wizard module of Schrodinger software. Protein crystals of TPI1 and PKM2 were preprocessed using the Protein Preparation Wizard module of the Schrodinger software. The nucleotide protein docking module was used to perform molecular docking analysis. The number of ligand rotation probes was set to 70000 and the maximum poses returned to 30. The lower the docking score, the lower the binding free energy, suggesting higher binding stability. Interaction between nucleic acid and Protein is deemed to be highly stable if binding energy is < ‐1000 kcal mol^−1^. Finally, binding interfaces were visualized using PyMOL version 2.50.

### HDAC3 Activity Assay

HDAC3 activity was measured using a fluorometric HDAC3 Activity Assay kit (Sigma‐Aldrich, USA) according to the manufacturer's instructions. Briefly, HDAC activity was detected based on a 2‐step enzymatic reaction. The first step of the reaction was the deacetylation of the acetylated lysine side chain by the HDAC‐containing sample. Subsequently, cleavage of the deacetylated substrate with the Developer Solution released a free, highly fluorescent group. The measured fluorescence positively correlated with the deacetylation activity of the samples.

### Immunoprecipitation (IP) and RNA Immunoprecipitation (RIP)

For the IP assay, the indicated cells were lysed in an IP lysis buffer. Then, the lysates were incubated with 10 µg of the corresponding antibodies overnight on a rotator at 4  °C. Ten microliters of protein A agarose beads were then added to the samples and incubated at 4 °C with gentle shaking for 3 h. After incubation, the bead‐protein mixtures were centrifuged and washed 3 times with the lysis buffer. The immunoprecipitated samples were further analyzed by WB.

RIP was performed using the Magna RIP RNA‐Binding Protein Immunoprecipitation Kit (Millipore, USA) according to the manufacturer's instructions. Briefly, the cell lysates containing RNase and protease inhibitors were centrifuged. Then, the cell lysates were incubated with magnetic beads coated with the indicated antibodies at 4 °C overnight. Proteinase K was used to treat the bead‐bound immunocomplexes at 55 °C for 30 min after washing with RIP wash buffer. Samples were then centrifuged and placed on a magnetic separator to isolate RNAs. The RNA fraction precipitated by RIP was analyzed by qPCR. RIP‐PCR products were detected at 4.8% AGE. The antibodies used in RIP assays are listed in Table S2 (Supporting Information).

### Chromatin Immunoprecipitation (ChIP) Assay

A Millipore EZ‐Magna ChIP kit (Catalog # 17–371) was used to perform all ChIP experiments. Briefly, 2 × 10^5^ cells were crosslinked with 1% formaldehyde for 10 min at room temperature. Next, 10 m glycine quenched cross‐linking. Chromatin was sonicated to 200–1000 bp in lysis buffer and ChIP DNA was extracted following the manufacturer's protocol. The antibodies used in this study are listed in Table S2 (Supporting Information).

### Immunohistochemistry (IHC)

The tissues were fixed in 4% paraformaldehyde for 24 48 h, dehydrated, and embedded in paraffin using a standard procedure. Paraffin sections (4 µm) were prepared in a three‐step process with a DAB staining kit (ZSGB‐BIO, China). Briefly, formalin‐fixed paraffin‐embedded tissue sections were incubated at 80 °C for 15 min, dewaxed in xylene, rinsed with graded ethanol, and rehydrated in double‐distilled water. For antigen retrieval, the slides were pretreated by steaming them in sodium citrate buffer for 15 min at 95 °C. The slices were then incubated with the indicated primary antibodies (Table S2, Supporting Information) at 4 °C overnight. The sections were then incubated with horseradish peroxidase‐labeled antimouse/antirabbit IgG secondary antibodies for 30 min. The slices were stained with DAB chromogen solution, incubated for 2 min, rinsed with PBS, and counterstained with hematoxylin. Multiplex immunohistochemistry was performed using a Multiplex Immunohistochemistry Kit (Absin, China) following the manufacturer's instructions.

### Ethics Approval and Consent to Participate

Human glioma tissues were obtained from patients treated at the Second Affiliated Hospital of Harbin Medical University. Fresh GBM tissues were collected from 14 patients via surgical resection (Department of Neurosurgery, Second Affiliated Hospital of Harbin Medical University) between 2020 and 2022. The inclusion criteria were as follows: patients who underwent surgical resection and pathological sampling; who were willing to provide disease‐related information/materials, including pathological diagnosis, relevant document records, medical records, and discarded tumor tissues from surgical resection; who were willing to provide contact information and maintain follow‐up; patients who did not participate in other clinical studies; who were willing (or their legal guardians were willing) to provide informed consent; and for whom the researcher believed that participating in this study would not affect the treatment effect. Patients who did not meet any of the above criteria were excluded from the study. Tumor tissues were stored by quick freezing until isolation of the patient‐derived GBM cell line. Informed consent was obtained from all patients enrolled in this study, and the Clinical Research Ethics Committee of the Second Affiliated Hospital of Harbin Medical University approved the study protocol (application number: KY2020‐070).

### Xenograft Model In Vivo

Six‐week‐old female specific pathogen‐free (SPF) BALB/c nude mice were purchased from the Animal Center of Beijing Vital River Laboratory Animal Technology and received mycoplasma testing followed by laboratory animal–a method for examination of Mycoplasma sp. A total of 1 × 10^6^ corresponding cells transfected with luciferase lentivirus resuspended in 6 µL PBS were stereotactically injected into the brain of each mouse at coordinates 1 mm anterior and 2 mm lateral to the right hemisphere relative to the bregma at a depth of 4 mm; this was followed by 7 days of tumor establishment. Mice were treated with TMZ (60 mg kg‐1 day‐1 per mouse) every 7 days. The intracranial tumors were measured using bioluminescence imaging. After all the mice died, their tissues were carefully extracted and fixed in 10% formalin for IHC staining. All procedures were approved by the Committee on the Ethics of Animal Experiments of Harbin Medical University. (approval number: SYDW2022‐043).

### Statistical Analysis

Significant differences between groups were identified using a two‐tailed Student's t‐test. The IC50 and tumor growth curves were analyzed using two‐way ANOVA, followed by Tukey's multiple comparison test. Overall survival curves were used to describe survival distributions, and the log‐rank test was used to assess statistical significance between different groups. Survival data were further analyzed using univariate and multivariate Cox regression analyses. Pearson's correlation coefficient was used to analyze the correlations between variables. USCS transcription factor enrichment analysis was performed using the DAVID website (http://david.abcc.ncifcrf.gov/home.jsp). Differential microarray analysis was performed using the R package limma and differential RNA‐seq analysis was performed using the R package DEseq2. ssGSEA was performed using the R package GSEAbase and GSEA was performed using the R package clusterProfiler. Gene sets were downloaded from GSEA_MSigDB (https://www.gsea‐msigdb.org/gsea/index.jsp). The extension packages pheatmap and ggplot2 were used to generate the figures. All R packages were run in R version 4.0.3. All results are expressed as the mean ± SD. All statistical analyses were performed using GraphPad software (version 7.0; GraphPad Software, CA, USA) or IBM SPSS Statistics (version 23.0; SPSS, Chicago, IL, USA). Bowtie2, Samtools, Deeptools, and MACS2 were used for ChIP‐seq analysis. Integrative Genomics Viewer was used to visualize the ChIP‐seq data. Statistical significance was set at *p* < 0.05. significance.

### Ethical Approval and Consent to Participate

Human glioma tissues were obtained from glioma patients at the Second Affiliated Hospital of Harbin Medical University. Informed consent was obtained from all patients enrolled in this study, and the Clinical Research Ethics Committee of the Second Affiliated Hospital of Harbin Medical University approved the study protocol. All animal experiments followed the protocols approved by the Institutional Committee on Animal Care of the Second Affiliated Hospital of Harbin Medical University.

### Consent for Publication

All authors agree to the publication.

## Conflict of Interest

The authors had no conflicts of interest.

## Author Contributions

C.Y. and Z.L. contributed equally: C.Y., Z.L., C.J., and J.C. designed the experiments. C.Y. and Z.L. performed the experiments. C.Y. and X.M. analyzed the data. C.Y. and J.C. wrote the manuscript. X.W. and D.S. contributed to the materials and analysis tools. T.X., K.T., P.S., J.Z., Y.S., W.M., and Y.L. participated in discussions regarding this work, and X.W. contributed to English language editing. All the authors have revised the manuscript accordingly.

## Supporting information



Supporting Information

## Data Availability

The data that support the findings of this study are available from the corresponding author upon reasonable request.
