# Supplementary material for: LncRNA‐Mediated TPI1 and PKM2 Promote Self‐Renewal and Chemoresistance in GBM
Source: Adv Sci (Weinh). 2024 Sep 28;11(44):2402600. doi: 10.1002/advs.202402600 (PMC11600202; doi:10.1002/advs.202402600)
Supplement: Supplementary file 1 — Supporting Information [file ADVS-11-2402600-s001.docx]

Supplementary Materials for LncRNA-mediated TPI1 and PKM2 promote self-renewal and chemoresistance in GBM

Changxiao Yang^1,2#^, Ziwei Li^1,4#^, Kaifu Tian^1^, Xiangqi Meng^1^, Xinyu Wang^1,2^, Dan Song^1,2^, Xuan Wang^5^, Tianye Xu^1,2^, Penggang Sun^1,2^, Junzhe Zhong^1,2^, Yu Song^1^, Wenbin Ma^1^, Yuxiang Liu^1^, Daohan Yu^1,2^, Ruofei Shen^1^, Chuanlu Jiang^1,3*^, Jinquan Cai^1*^

**Affiliations:**

^1^Department of Neurosurgery, The Second Affiliated Hospital of Harbin Medical University, Harbin 150086, China.

^2^Future Medical Laboratory, The Second Affiliated Hospital of Harbin Medical University, 150086, Harbin, China.

^3^The Sixth Affiliated Hospital of Harbin Medical University, Harbin 150086, China.

^4^Beijing Tiantan Hospital, Capital Medical University, 100070, Beijing, China

^5^Department of Neurosurgery, Union Hospital, Tongji Medical College, Huazhong University of Science and Technology, Wuhan, Hubei, China

***Corresponding author:** Email: caijinquan@hrbmu.edu.cn (J.C.); jiangchuanlu@hrbmu.edu.cn (C.J.)

#These authors contributed equally: Changxiao Yang, Ziwei Li.

**Supplementary Figures and Figure Legends**

**
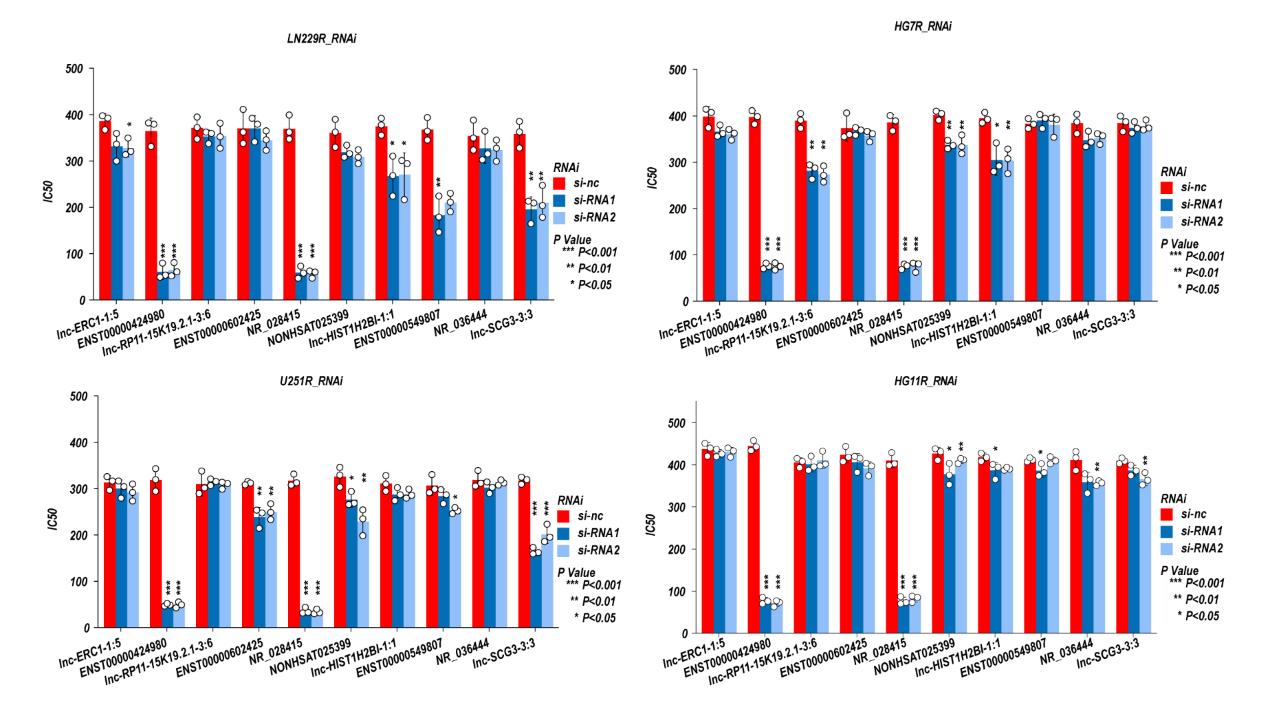
**

**Supplementary Fig 1. Screening of lncRNAs associated with TMZ resistance in TMZ-resistant GBM cells by RNAi assay.** Determination of TMZ IC50 after knockdown of the top 10 upregulated lncRNAs in TMZ-resistant GBM cells upon TMZ treatment (n = 3). Values presented as mean ± S.D. from three independent experiments were determined using the Student’s t-test. Significant results are presented as *, P < 0.05; **, P < 0.01; or ***, P < 0.001.


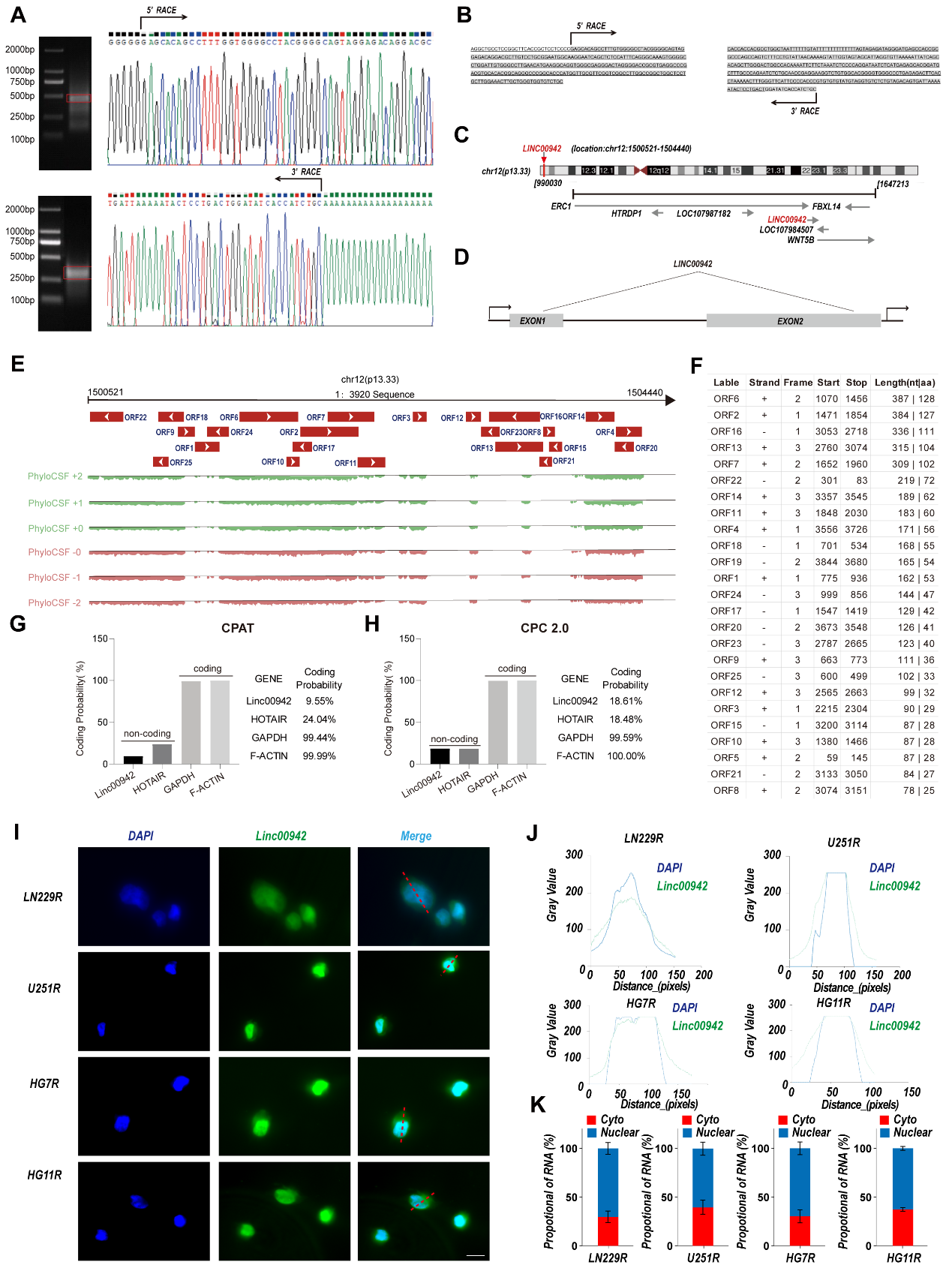


**Supplementary Fig 2.** **Analysis of the transcriptional profiles, coding potentials, and subcellular localization of Linc00942.**

**A.** Sequence of Linc00942 validated by RACE experiments. Left: agarose assay of PCR products from the 5’RACE or 3’RACE procedure. The major PCR product is marked. Right: The nucleotide sequence of Linc00942.

**B, C.** The transcriptional location of Linc00942.

**D.** The exon structure of Linc00942.

**E, F.** Prediction of putative proteins encoded by Linc00942 using PhyloCSF and ORF finder analyses.

**G, H.** Coding potential analysis of Linc00942 using CPAT and CPC 2.0. The lncRNA HOTAIR was used as a noncoding gene control. GAPDH and F-actin were used as coding gene controls.

**I, J.** FISH assay and colocalization analysis of Linc00942 in the indicated TMZ-resistant GBM cells. Representative images are shown, and cells used for colocalization analysis are marked, scale bar = 50 μm.

**K.** qRT-PCR analysis of the cytoplasmic to nuclear ratio of Linc00942 in TMZ-resistant cells (n=3). Values in (K) represent the mean ± SD from three independent experiments.


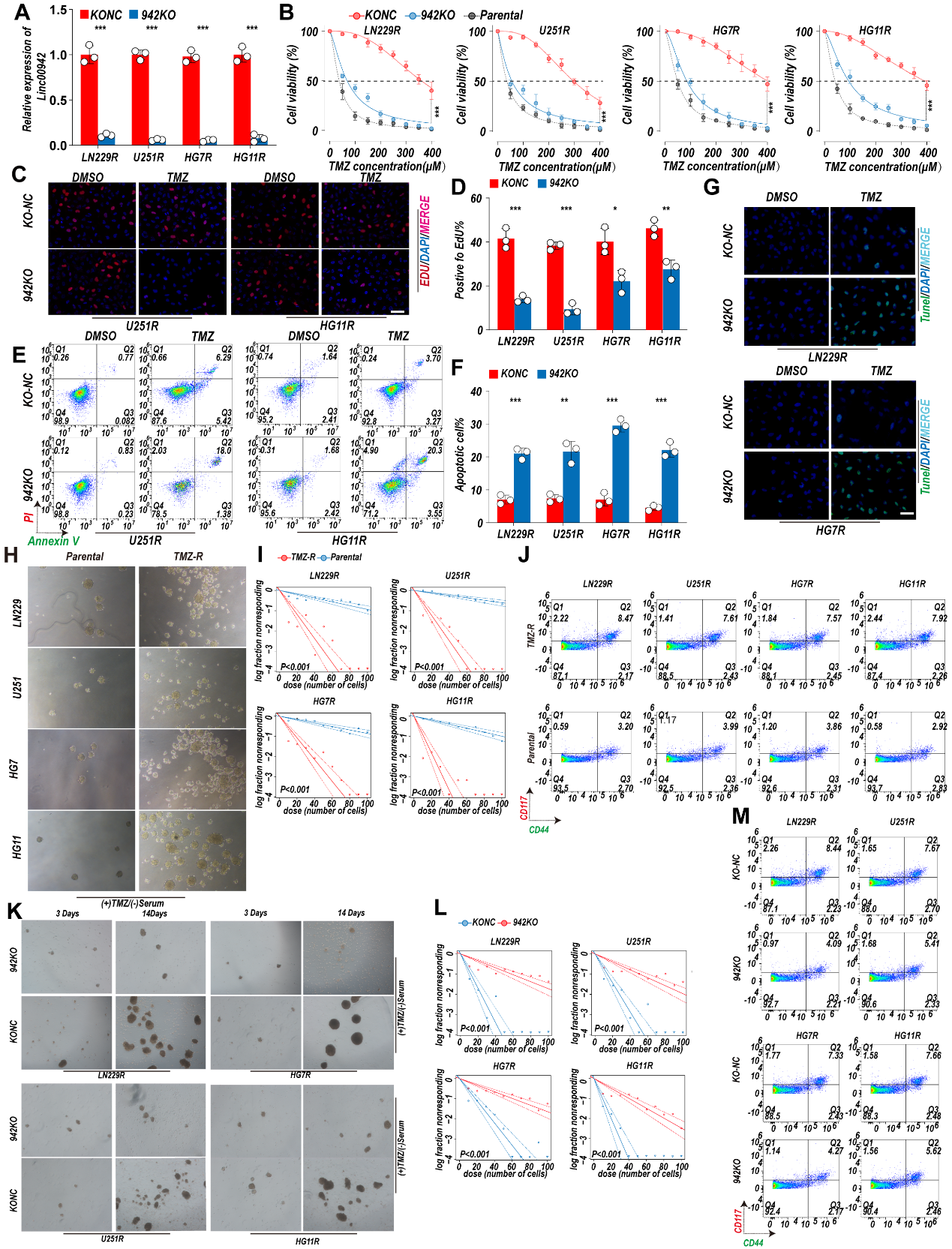


**Supplementary Fig 3. Knockdown of Linc00942 restores TMZ sensitivity and inhibits self-renewal among TMZ-resistant GBM cells.**

**A.** Construction of Linc00942-knockout TMZ-resistant GBM cells validated by qRT-PCR (n = 3).

**B.** IC50 of TMZ in LN229R, U251R, HG7R, and HG11R cells transfected with KONC or 942 KO cells. Corresponding parental cells were used as controls (n = 3).

**C, D.** EdU assay of the indicated cells treated with DMSO or TMZ (100 μM) for 72 h (n = 3), Scale bar = 50 μm.

**E, F.** Apoptosis rate detected by flow cytometry in the indicated cells treated with DMSO or TMZ (200 μM) for 72 h (n = 3)

**G**. TUNEL assay of the indicated cells treated with DMSO or TMZ (200 μM) for 72 h, Scale bar = 50 μm.

**H.** Sphere formation assay of TMZ-resistant GBM cells and corresponding parental GBM cells cultured in serum-free medium with TMZ (100 μM) on an ultralow attachment multiwell plate for 14 d.

**I**. ELDA assay of TMZ-resistant GBM cells and corresponding parental GBM cells cultured in serum-free medium with TMZ (100 μM) on an ultralow attachment multiwell plate for 14 d.

**J.** Stem cell markers detected by flow cytometry assays of TMZ-resistant GBM cells and corresponding parental GBM cells cultured in serum-free medium with TMZ (100 μM) for 72 h

**K.** Sphere formation assay of TMZ-resistant GBM cells transfected with KONC or 942KO cultured in a corresponding serum-free medium with TMZ (100 μM) on an ultralow attachment multiwell plate for 3 d and 14 d.

**L**. ELDA assay of TMZ-resistant GBM cells transfected with KONC or 942KO cultured in serum-free medium with TMZ (100 μM) on an ultralow attachment multiwell plate for 14 d.

**M.** Stem cell markers detected by flow cytometry assays of T TMZ-resistant GBM cells transfected with KONC or 942KO cultured in serum-free medium with TMZ (100 μM) for 72 h. Values in (A, B, D, F, H, and K) represent the mean ± SD from three independent experiments. P-values (A, D, and F) were determined using a two-tailed Student’s t-test. P-values in (B) were determined using two-way ANOVA, followed by Tukey’s multiple comparison test. P-values for (H and K) were determined using ELDA. Significant results are presented as *, P < 0.05; **, P < 0.01; or ***, P < 0.001.


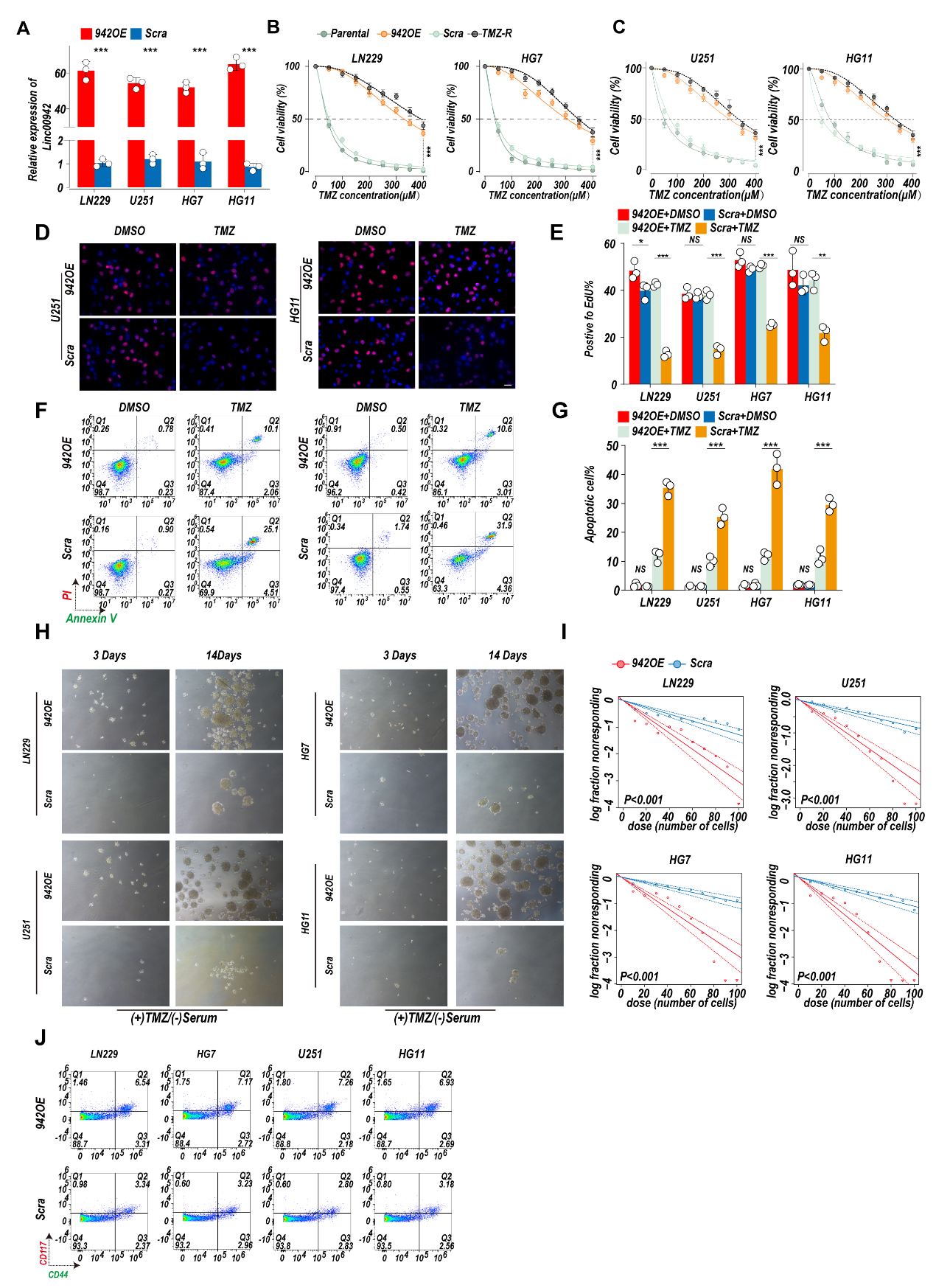


**Supplementary Fig 4. Overexpression of Linc00942 promotes TMZ resistance and self-renewal in GBM cells.**

**A.** Construction of Linc00942-overexpressing GBM cells validated by qRT‒PCR (n = 3).

**B, C.** IC50 analysis of TMZ in Linc00942-overexpressing LN229, U251, HG7, and HG11 cells. Corresponding scrambled cells were used as controls (n = 3).

**D, E.** EdU assay of cells treated with DMSO or TMZ (100 μM) for 72 h (n = 3). Scale bar = 50 μm.

**F, G.** Apoptosis rate detected by flow cytometry in the indicated cells treated with DMSO or TMZ (200 μM) for 72 h (n = 3).

**H.** Sphere formation assay of Linc00942-overexpressing and scramble GBM cells cultured in corresponding serum-free medium with TMZ (100 μM) on an ultralow attachment multiwell plate for 3 d and 14 d.

**I**. ELDA assay of indicated cells cultured in serum-free medium with TMZ (100 μM) on an ultralow attachment multiwell plate for 14 d.

**J.** Stem cell markers detected by flow cytometry assays of indicated cells cultured in serum-free medium with TMZ (100 μM) for 72 h. Values in (A, B, C, E, G, and I) represent the mean ± SD from three independent experiments. P-values (A, E, and G) were determined using a two-tailed Student’s t-test. P-values in (B and C) were determined using two-way ANOVA, followed by Tukey’s multiple comparison test. P-values in (I) were determined using ELDA. Significant results are presented as** P < 0.01, *** P < 0.001.


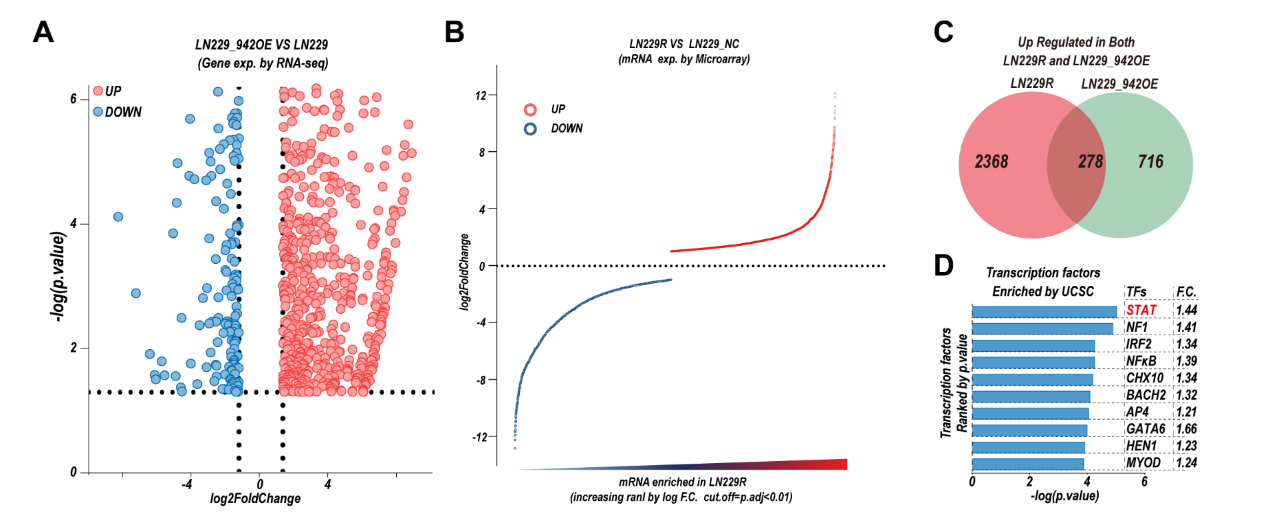


**Supplementary Fig 5. Transcriptomic data reveal that coupregulated genes in Linc00942-overexpressing and TMZ-resistant GBM cells LN229R are enriched in the STAT target.**

**A.** Volcano plot showing genes that were significantly differentially expressed in Linc00942-overexpressing and scramble LN229 cells, as determined by RNA-Seq. Red: upregulated genes; blue: downregulated genes. Cut off: log2Foldchange ≥ 1, P-value < 0.05.

**B.** Gene ranking plot showing mRNAs that were significantly differentially expressed in TMZ-resistant and parental LN229 cells, as determined by microarray. Red: upregulated genes; blue: downregulated genes. Cut off: log2Foldchange ≥ 1, p.adj < 0.01.

**C.** Venn diagram showing coupregulated genes in Linc00942-overexpressing and TMZ-resistant LN229 cells.

**D.** Transcription factor enrichment analysis of coupregulated genes in Linc00942-overexpressing and TMZ-resistant LN229 cells by UCSC.


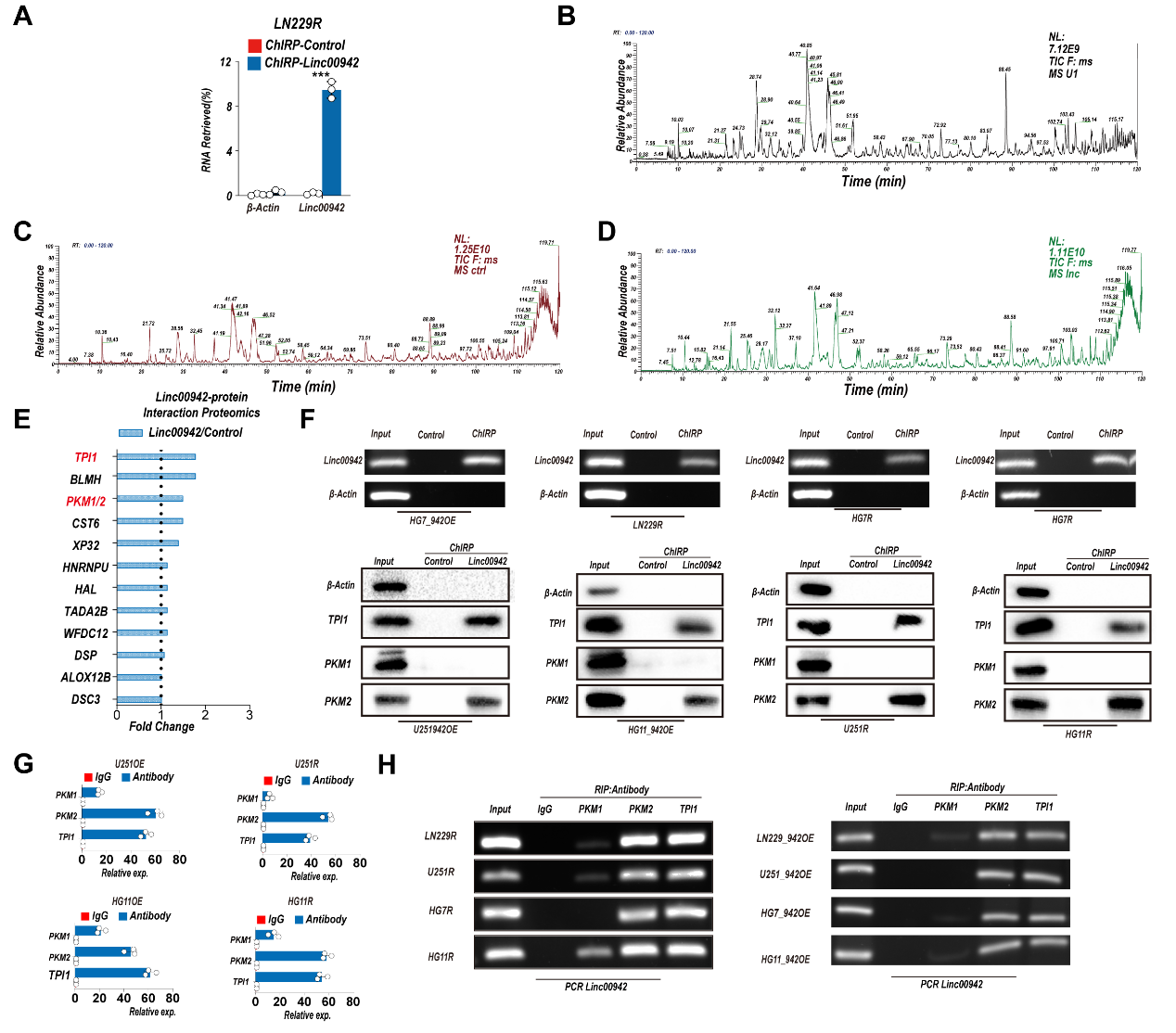


**Supplementary Fig 6. Identification of Linc00942-interacting proteins.**

**A.** Specific biotin-labeled Linc00942 probes were used to capture Linc00942 from total cellular extracts of TMZ-resistant GBM LN229R cells (n = 3). Control probes and β-actin were used as controls.

**B, C, D** Quality control of samples isolated using the corresponding probes. lncRNA U1 served as a positive control (B) and scrambled oligos served as a negative control (C). Peptides were isolated using ChIRP (D).

**E.** Significantly enriched proteins in Linc00942 probes.

**F.** ChIRP-WB showed that TPI1 and PKM2 were Linc00942-interacting proteins in Linc00942-overexpressing and U251R and HG11R cells. An agarose gel assay was used for quality control.

**G, H** RIP, followed by qPCR and PCR of Linc00942 using the indicated antibodies and specific primers (n = 3). Values in (A and G) represent the mean ± SD from three independent experiments. P-values in (A) were determined using a two-tailed Student’s t-test. Significant results are presented as *** P < 0.001.


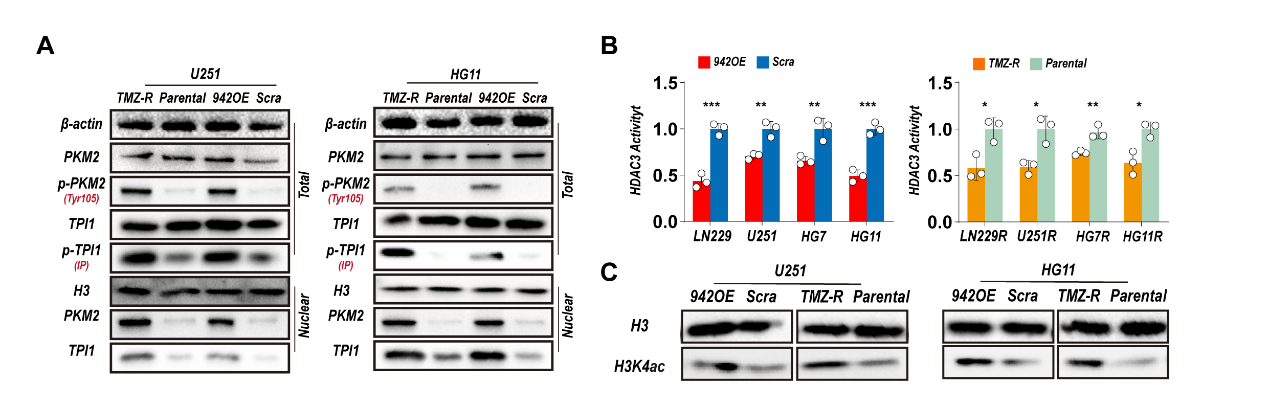


**Supplementary Fig 7. Phosphorylation and nuclear translocation of TPI1 and PKM2 were promoted in Linc00942-overexpressing and TMZ-resistant U251 and HG11 cells.**

**A.** WB analysis of Linc00942 binding factors, TPI1 and PKM2. The total expression, phosphorylation, and nuclear translocation levels of both proteins were detected. A pan-phospho antibody was used to detect the phosphorylation of TPI1 by IP.

**B.** HDAC3 activity assay performed in the indicated cells (n = 3).

**C.** WB assay of H3K4ac in the indicated U251 and HG11 cells. Values in (B) represent the mean ± SD from three independent experiments. P-values in (B) were determined by a two-tailed Student’s t test. Significant results are presented as *, P < 0.05; **, P < 0.01; or ***, P < 0.001.


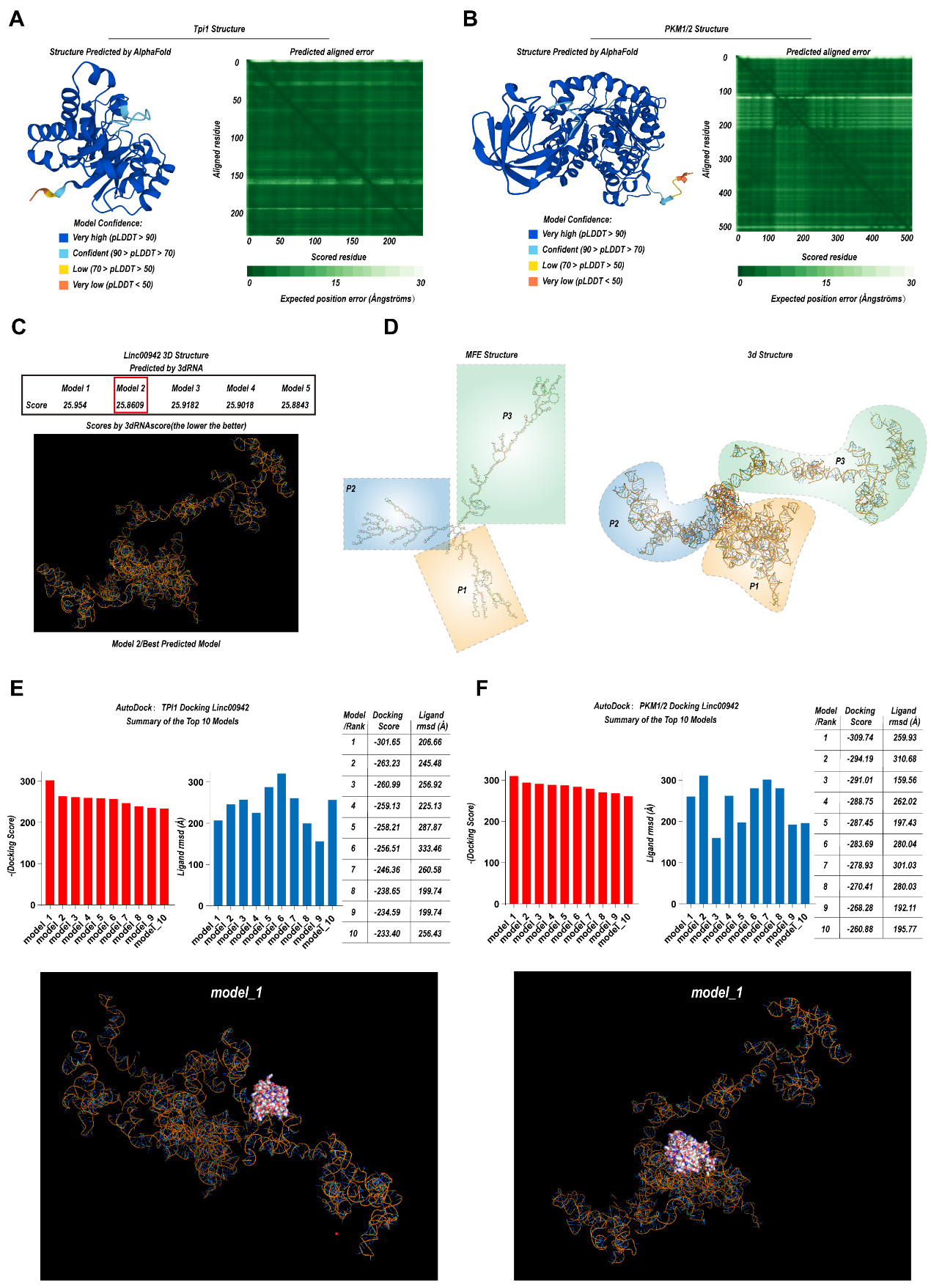


**Supplementary Fig 8. Construction of models of Linc00942 interacting with TPI1 and PKM2**

**A, B.** Structure information of TPI1 and PKM2.

**C.** Top five 3D structures of Linc00942. The best model was marked and visualized using the PyMol software.

**D.** Schematic diagram of the three major substructures of Linc00942, marked by different colors and named P1, P2, and P3. Both MFE and 3d structures are shown.

**E, F.** Docking score and ligand RMSD of the top 10 models of the Linc00942 interacting with TPI1 and PKM2. The best docking model for each protein has been presented.


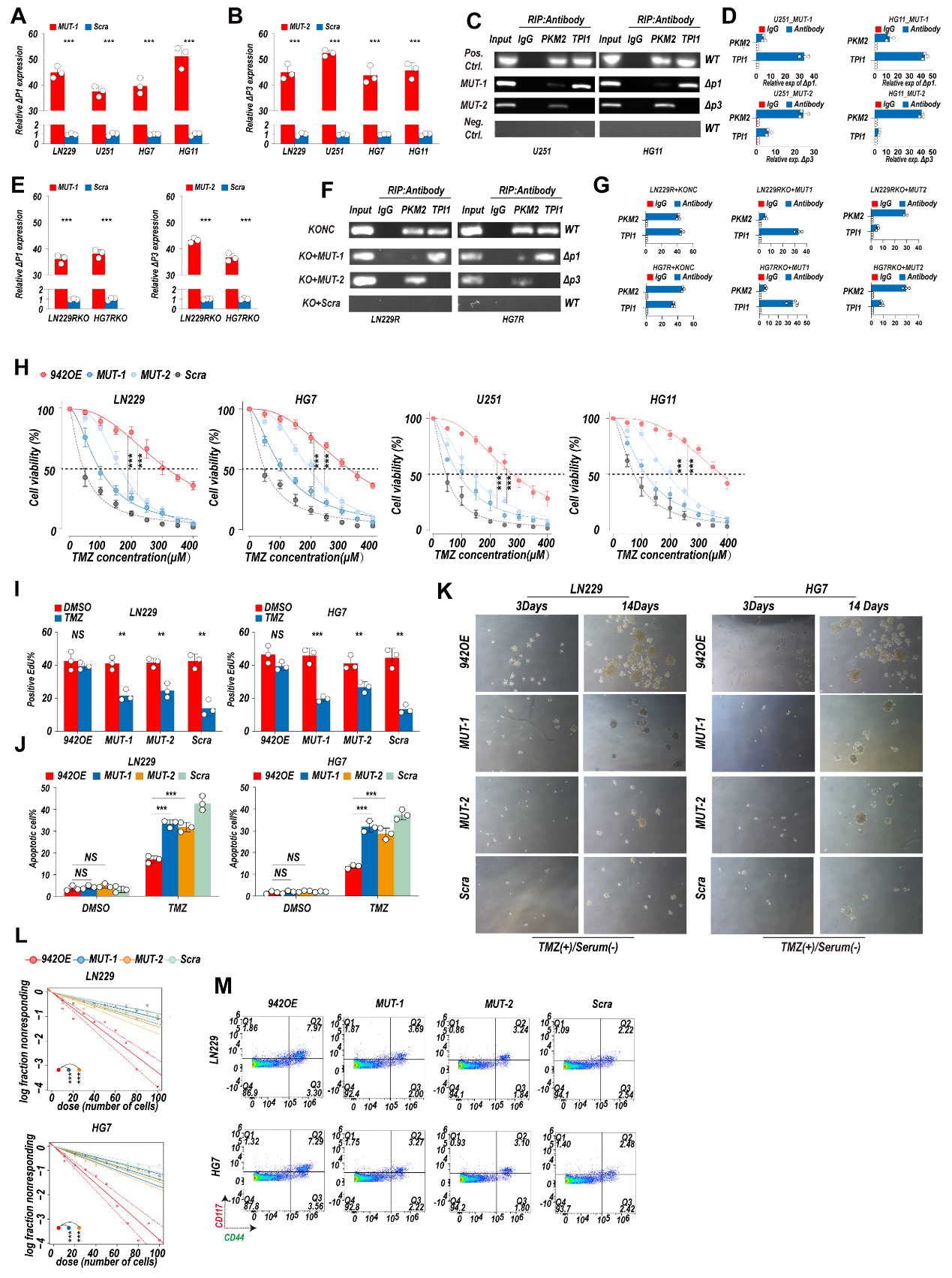
**Supplementary Fig 9. Increased TMZ tolerance and self-renewal capacity were not induced by mutant Linc00492.**

**A.** Construction of MUT-1 GBM cells through transfection of the indicated cells with the ΔP1 plasmid, validated by qPCR (n = 3).

**B.** Construction of MUT-2 GBM cells through transfection of the indicated cells with the ΔP3 plasmid, validated by qPCR (n = 3).

**C, D.** RIP assay followed by PCR and q-PCR (n = 3) performed in the indicated cells using specific TPI1 or PKM2 antibodies and specific primers to detect Linc00942 ΔP1 or ΔP3.

**E.** Construction of MUT-1 or MUT-2 GBM cells through transfection of the indicated Linc00942 knockout TMZ-resistant cells with the ΔP1 plasmid, validated by qPCR (n = 3).

**F, G.** RIP assay followed by PCR and q-PCR (n = 3) performed in the indicated cells using specific TPI1 or PKM2 antibodies and specific primers to detect Linc00942 ΔP1 or ΔP3.

**H.** IC50 analysis of TMZ in the indicated cells, corresponding to scrambled control cells (n = 3).

**I.** EdU assay of cells treated with DMSO or TMZ (100 μM) for 72 h (n = 3). Scale bar = 10 μm.

**J.** Apoptosis rate detected by flow cytometry in the indicated cells treated with DMSO or TMZ (200 μM) for 72 h (n = 3). Scale bar = 10 μm.

**K.** Sphere formation assay for the indicated cells cultured in serum-free medium with TMZ (100 μM) on an ultralow attachment multiwell plate for 3 d and 14 d. Representative images are shown.

**L**. ELDA assay of indicated cells cultured in serum-free medium with TMZ (100 μM) on an ultralow attachment multiwell plate for 14 d.

**M.** Stem cell markers detected by flow cytometry assays of indicated cells cultured in serum-free medium with TMZ (100 μM) for 72 h. Values in (A, B, D, E, F, G, H, I, J, and L) represent the mean ± SD from three independent experiments. P-values (A, B, E, I, and J) were determined using a two-tailed Student’s t-test. P-values in (H) were determined using two-way ANOVA, followed by Tukey’s multiple comparison test. P-values in (L) were determined using ELDA. Significant results are presented as **, P < 0.01 and ***, P < 0.001.


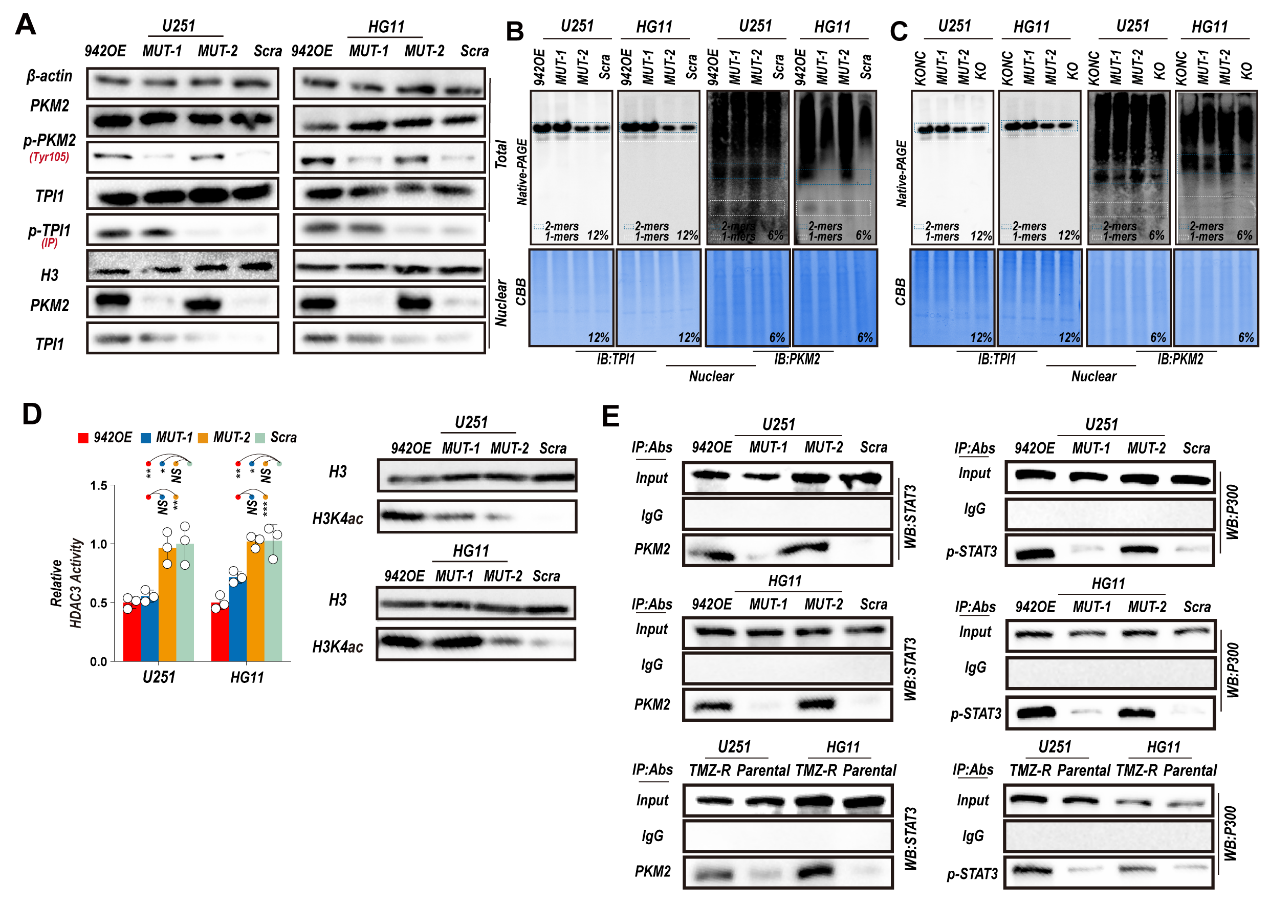


**Supplementary Fig 10. Linc00942 promoted the phosphorylation, dimerization, and nuclear translocation of TPI1 and PKM2 in U251 and HG11 cells by direct interaction.**

**A.** WB analysis of the Linc00942 binding factors TPI1 and PKM2 in the indicated cells. The total expression, phosphorylation, and nuclear translocation levels of both proteins were detected. A pan-phospho antibody was used to detect the phosphorylation of TPI1 by IP.

**B, C.** Native PAGE analysis of TPI1 and PKM2 polymers in the indicated cells.

**D.** HDAC3 assay (n = 3) and WB for acetylation modifications in the indicated cells.

**E.** IP assay of the interaction between PKM2/STAT3 and STAT3/p300 in the indicated cells. Values in (D) represent the mean ± SD from three independent experiments. P-values in (D) were determined using a two-tailed Student’s t-test. Significant results are presented as *, P < 0.05; or **, P < 0.01; or ***, P < 0.001.


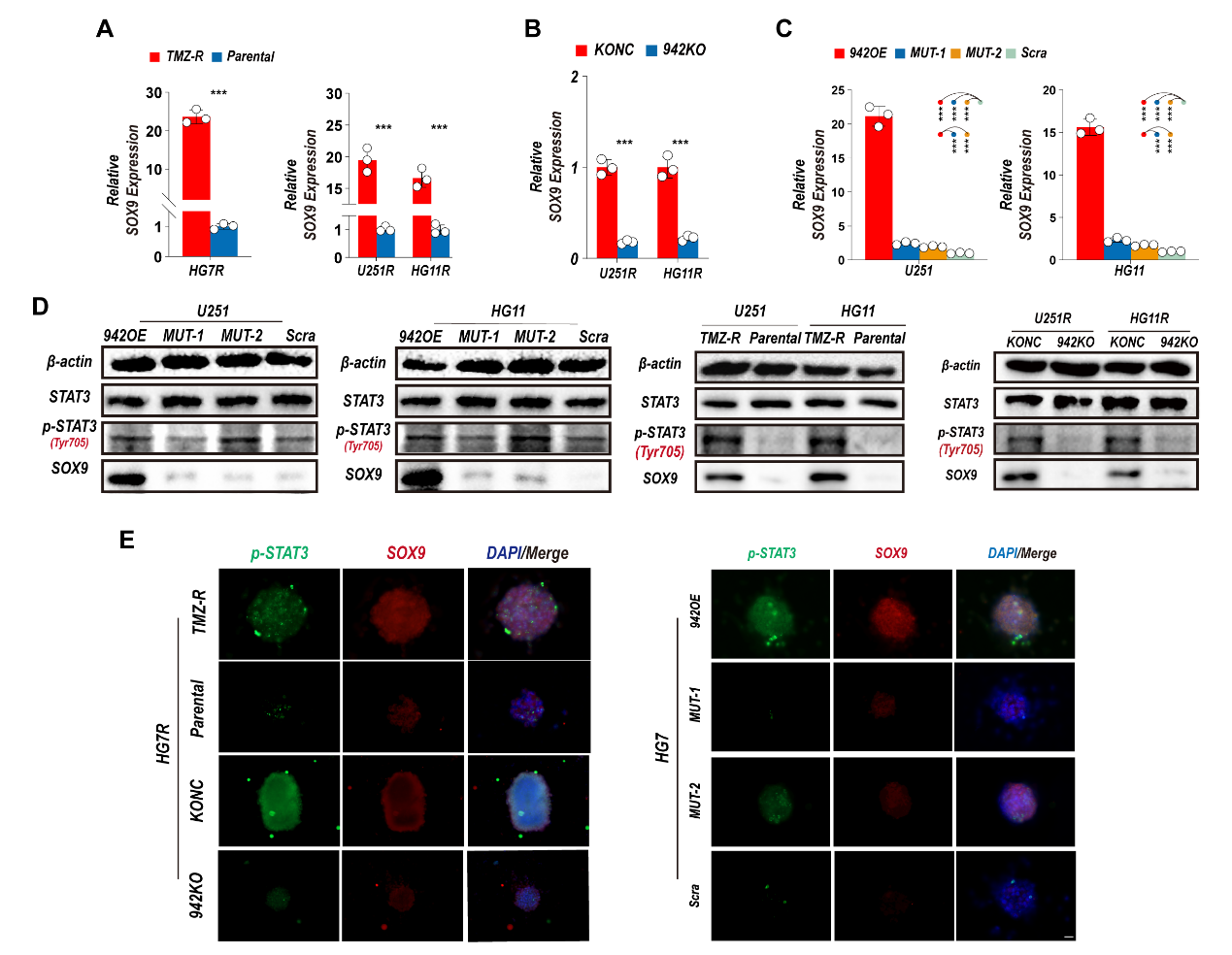


**Supplementary Fig 11. Linc00942 promoted the phosphorylation, dimerization, and nuclear translocation of TPI1 and PKM2 in U251 and HG11 cells by direct interaction.**

**A-C.** q-PCR analysis of SOX9 in the indicated cells with specific primers (n = 3).

**D.** WB assay of STAT3, p-STAT3, and SOX9 in the indicated cells.

**E.** Sphere-IF assay of p-STAT3 and SOX9 in the indicated cells. Scale bar = 50 μm. Values in (A, B, and C) represent the mean ± SD from three independent experiments. P-values in (A, B, and C) were determined by a two-tailed Student’s t test. Significant results are presented as **, P < 0.01 or ***, P < 0.001.


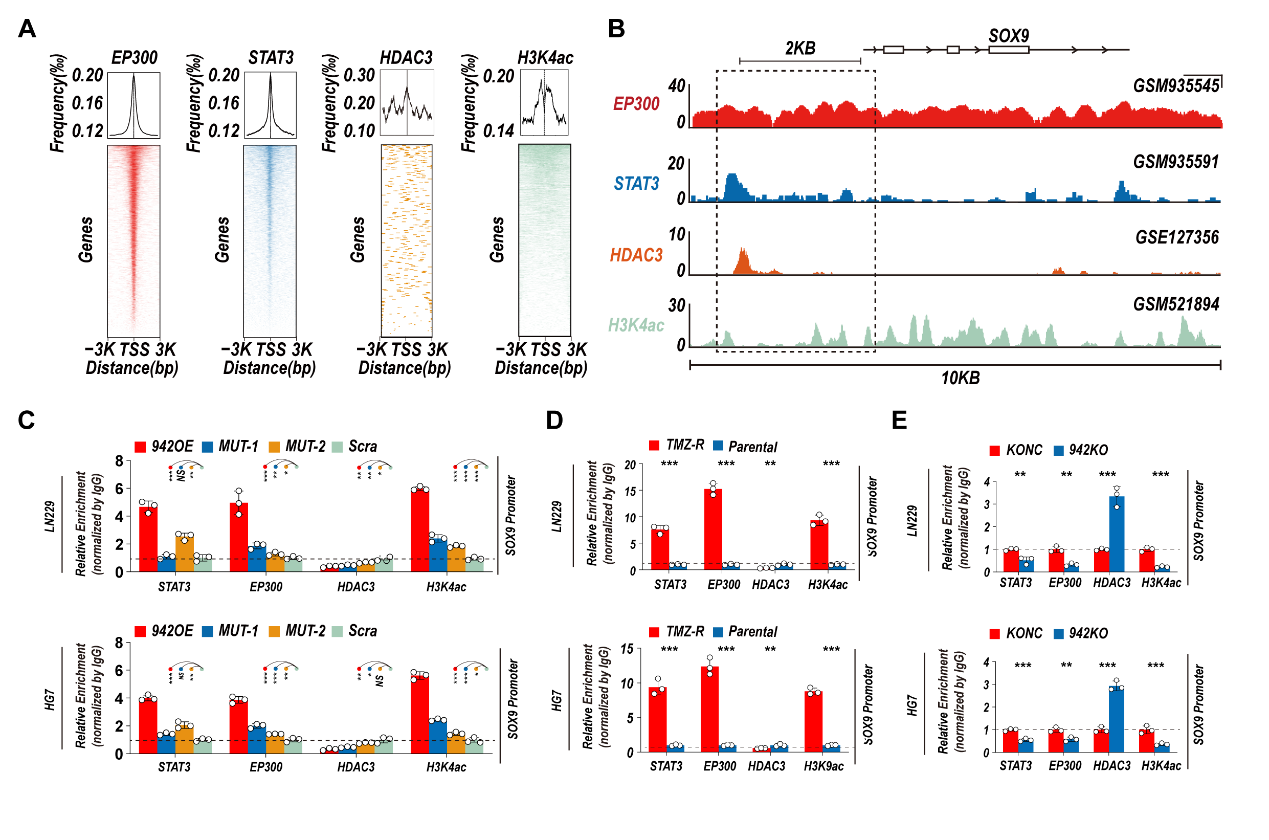


**Supplementary Fig 12. ChIP analysis showed that Linc00942 promoted acetylation of the promoter region of SOX9 at H3K4.**

**A.** Heatmap of EP300, STAT3, HDAC3, and H3K4ac binding sites within a region spanning ± 3 kb around the transcriptional start sites in the whole genome.

**B.** ChIP-seq analysis showing the signal peaks of EP300, STAT3, HDAC3, and H3K4ac in the promoter regions of the SOX9 gene.

**C.** ChIP‒qPCR analysis using specific p-STAT3, EP00, HDAC3, and H3K4ac antibodies and specific primers to detect the SOX9 promoter in the indicated cells (n = 3).

D. ChIP-qPCR analysis using specific p-STAT3, EP00, HDAC3, and H3K4ac antibodies and specific primers to detect the SOX9 promoter in TMZ-resistant and parental LN229 and HG7 cells (n = 3).

**E.** ChIP-qPCR analysis using specific p-STAT3, EP00, HDAC3, and H3K4ac antibodies and specific primers to detect the SOX9 promoter in TMZ-resistant LN229R and HG7R cells transfected with KONC or 942KO (n = 3). Values in (C, D, and E) represent the mean ± SD from three independent experiments. P-values in (C, D, and E) were determined by a two-tailed Student’s t test. Significant results are presented as *, P < 0.05; or **, P < 0.01; or ***, P < 0.001.


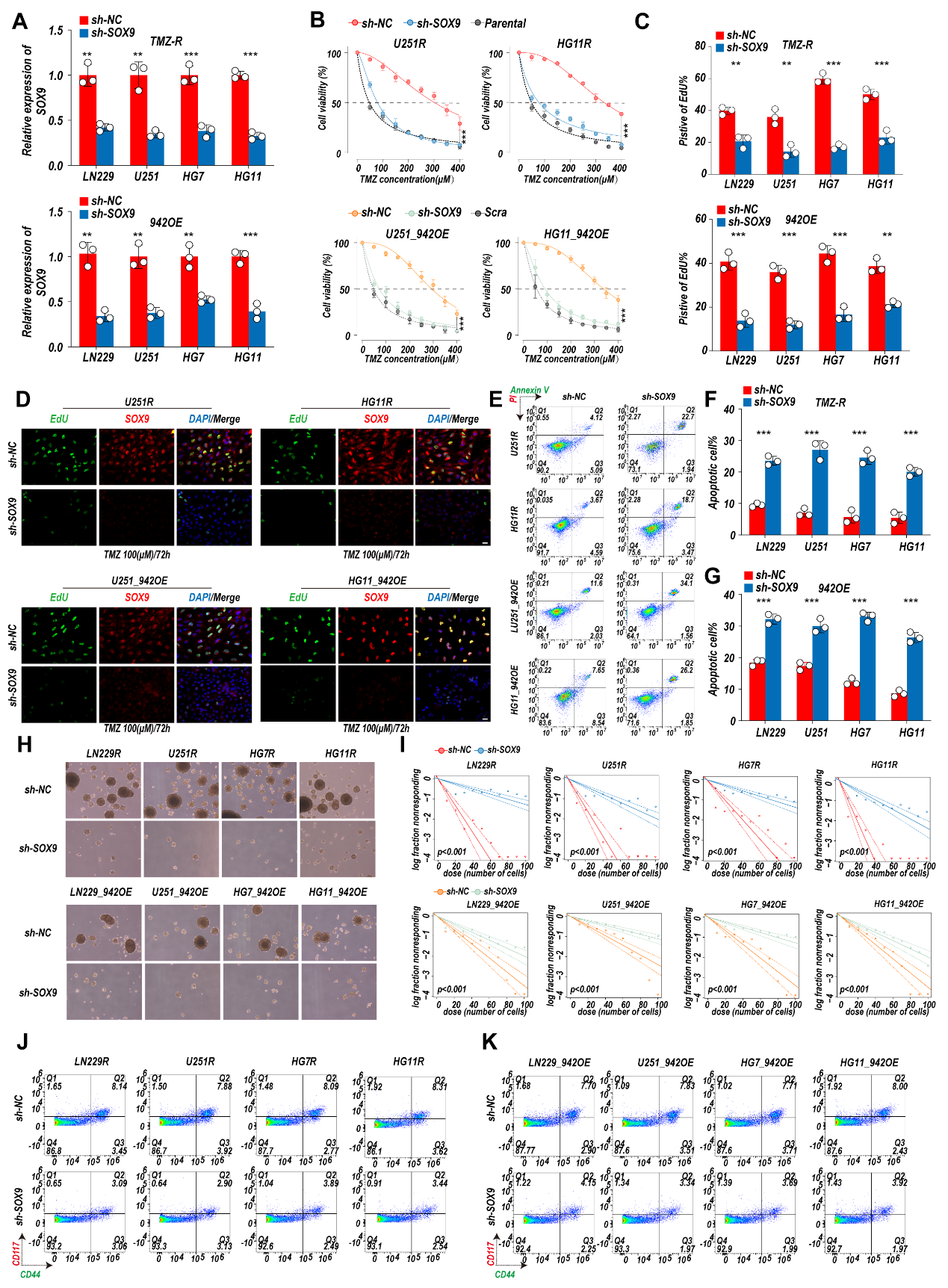


**Supplementary Fig 13. Knockdown of SOX9 inhibited TMZ resistance and self-renewal in Linc00942-overexpressing and TMZ-resistant cells.**

**A.** q-PCR analysis of the indicated cells transfected with sh-NC or sh-SOX9 with specific primers (n = 3).

**B.** IC50 of TMZ detected in the indicated cells transfected with sh-NC or sh-SOX9. Corresponding scramble or parental cells were used as controls (n = 3).

**C, D.** EdU assay of the indicated cells transfected with sh-NC or sh-SOX9 treated with DMSO or TMZ (100 μM) for 72 h (n = 3), scale bar = 50 μm. Representative images and the average number of EdU-positive cells are shown.

**C, D.** EdU assay of the indicated cells transfected with sh-NC or sh-SOX9 treated with DMSO or TMZ (100 μM) for 72 h (n = 3), scale bar = 50 μm.

**E-G.** Apoptosis rate detected by flow cytometry of cells treated with DMSO or TMZ (200 μM) for 72 h (n = 3)

**H.** Sphere formation assay for the indicated cells transfected with sh-NC or sh-SOX9 cultured in the corresponding serum-free medium with TMZ (100 μM) on an ultralow attachment multiwell plate for 14 d. Representative images are shown.

**I**. ELDA assay of indicated cells cultured in serum-free medium with TMZ (100 μM) on an ultralow attachment multiwell plate for 14 d.

**J, K.** Stem cell markers detected by flow cytometry assays of indicated cells cultured in serum-free medium with TMZ (100 μM) for 72 h. Values in (A, B, C, F, G, and I) represent the mean ± SD from three independent experiments. P-values (A, C, F, and G) were determined using a two-tailed Student’s t-test. P-values in (B) were determined using two-way ANOVA, followed by Tukey’s multiple comparison test. P-values in (I) were determined using ELDA. Significant results are presented as ** P < 0.01 or ***, P < 0.001.


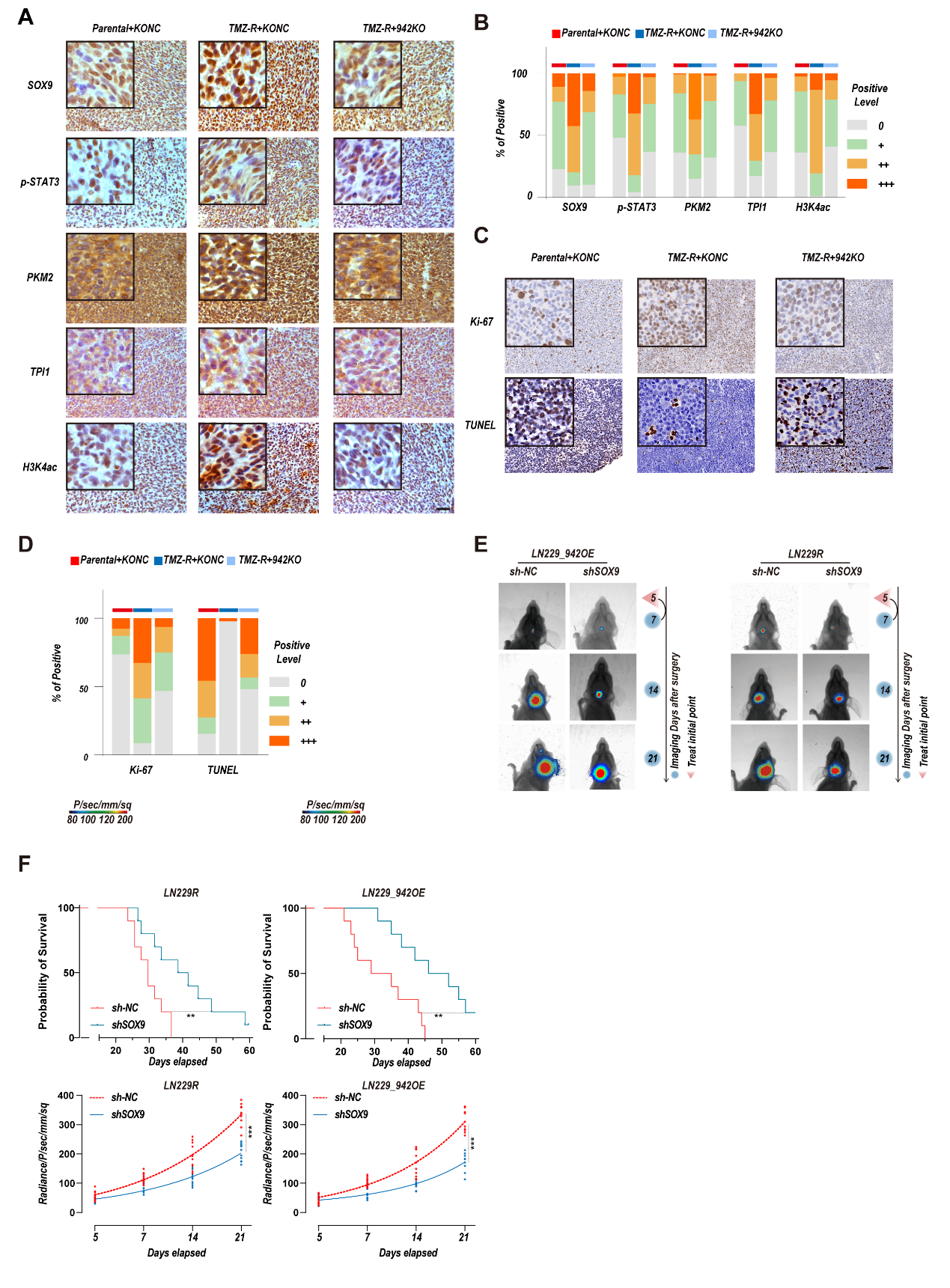


**Supplementary Fig 14. *In vivo* experiments validated that the knockdown of SOX9 inhibited TMZ resistance in Linc00942-overexpressing and TMZ-resistant cells.**

**A, B.** IHC staining of SOX9, TPI1, PKM2, and H3K4AC in consecutive brain sections of orthotopic GBM mouse models bearing TMZ-resistant LN229R cells transfected with KONC or 942KO and parental LN229 cells transfected with KONC as a control. The histogram represents a quantitative evaluation of the IHC assay. Scale bar = 50 μm.

**C-D**. IHC staining of KI67 and TUNEL assay in consecutive brain sections of orthotopic GBM mouse models bearing TMZ-resistant LN229R cells transfected with KONC or 942KO, and parental LN229 cells transfected with KONC as a control. The histogram represents a quantitative evaluation of the IHC assay. Scale bar = 50 μm.

**E**. Bioluminescence images of mice bearing tumors derived from Linc00492-overexpressing or TMZ-resistant LN229 cells transfected with sh-NC or sh-SOX9. The mice were then treated with TMZ.

**F**. Quantification of bioluminescence curves (top) and Kaplan‒Meier survival curves (bottom) of mice with Linc00492-overexpressing or TMZ-resistant LN229 cells transfected with sh-NC or sh-SOX9 (n = 10). P-values in (D) were determined by the Log-rank Test (top) and two-way ANOVA, followed by Tukey’s multiple comparison test (bottom). Significant results are presented as ** P < 0.01 or ***, P < 0.001.


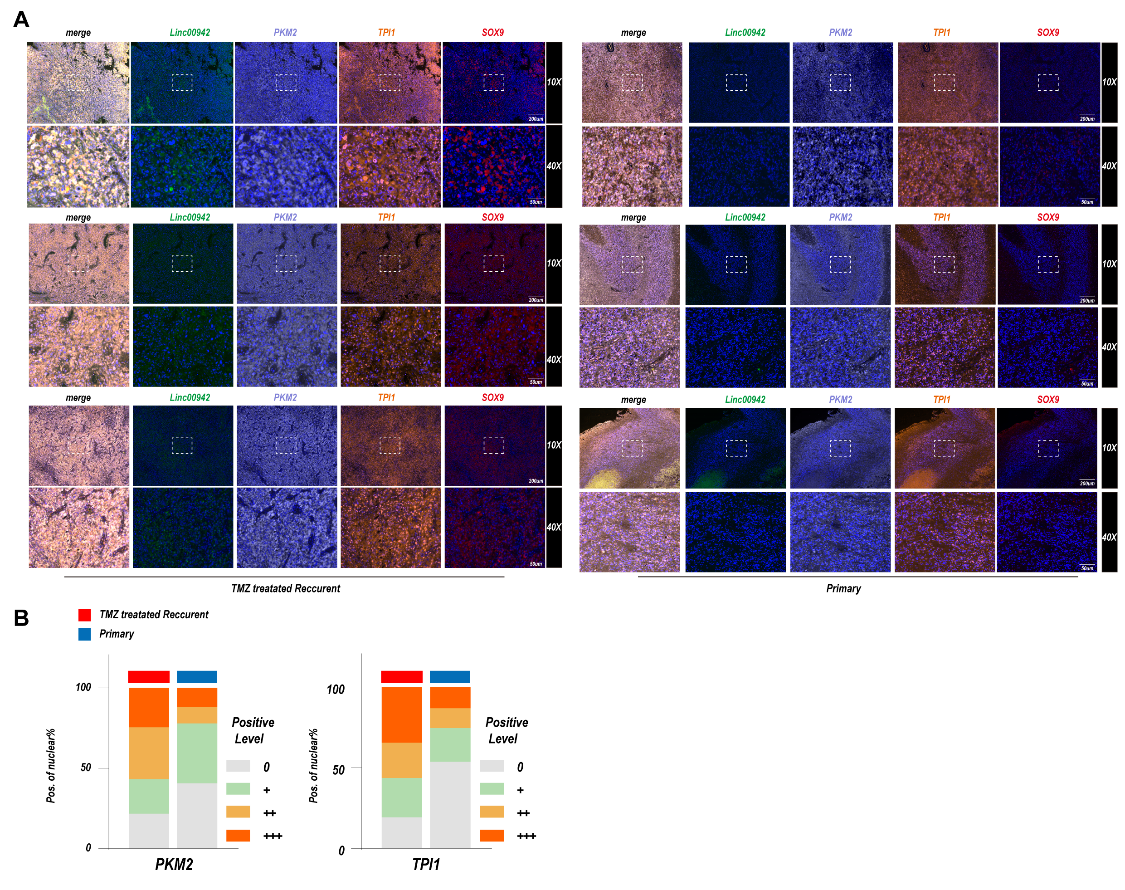


**Supplementary Fig 15. FISH combined with mIHC assay validated that Linc00942 was increased in TMZ-treated recurrent GBM samples, promoting nuclear translocation of TPI1 and PKM2 and expression of SOX9.**

**Supplementary Tables**

**Supplementary Table S1. Significant specific binding proteins of Linc00942 identified through ChIRP-MS.**

| Gene names | Number of proteins | Unique peptides | Sequence coverage | MS count | Q-value | Scores | iBAQ |
| --- | --- | --- | --- | --- | --- | --- | --- |
| XP32 | 1 | 6 | 25.6 | 10 | 0 | 57.517 | 2.12E+07 |
| WFDC12 | 1 | 2 | 23.4 | 2 | 0 | 11.918 | 6.06E+05 |
| A2ML1 | 4 | 4 | 3.1 | 6 | 0 | 21.806 | 9.05E+04 |
| CST6 | 1 | 2 | 14.1 | 3 | 0 | 12.171 | 2.61E+06 |
| DSC1 | 2 | 11 | 18.5 | 47 | 0 | 204.44 | 2.63E+06 |
| DSC3 | 2 | 3 | 4.8 | 8 | 0 | 37.571 | 3.04E+05 |
| DSP | 3 | 87 | 31.8 | 155 | 0 | 323.31 | 9.62E+06 |
| ENO1 | 21 | 4 | 12.2 | 9 | 0 | 29.653 | 1.41E+06 |
| ALOX12B | 2 | 5 | 8 | 8 | 0 | 32.987 | 1.20E+06 |
| GAPDH | 4 | 8 | 33.8 | 27 | 0 | 109.51 | 9.24E+06 |
| HAL | 5 | 2 | 14.6 | 2 | 0 | 10.763 | 6.89E+05 |
| HNRNPU | 16 | 11 | 14.2 | 19 | 0 | 93.318 | 2.63E+06 |
| KPRP | 1 | 22 | 49.1 | 96 | 0 | 197.16 | 2.57E+07 |
| PKM | 13 | 6 | 18.6 | 9 | 0 | 51.905 | 4.94E+05 |
| BLMH | 4 | 4 | 21.6 | 6 | 0 | 28.792 | 1.07E+06 |
| TGM1 | 5 | 14 | 18.7 | 26 | 0 | 107.93 | 2.22E+06 |
| TGM3 | 2 | 16 | 27.9 | 32 | 0 | 141.93 | 3.37E+06 |
| TPI1 | 5 | 4 | 20.1 | 4 | 0 | 35.434 | 7.99E+05 |

**Supplementary Table S2. Primary antibodies used in the present study.**

| Antigens | Manufacturer | Catalog numbers | Application |
| --- | --- | --- | --- |
| PKM2 | Proteintech Group | 60268-1-Ig | 1:1000 for WB, 1:200 for IF, |
|  |  |  | 1:100 for IHC and mIHC, 1:50 for RIP and IP |
| p-PKM2 | Affinity | #DF2975 | 1:1000 for WB, |
| TPI1 | Proteintech Group | 10713-1-Ig | 1:3000 for WB, 1:500 for IF, |
|  |  |  | 1:200 for IHC and mIHC, 1:100 for RIP and IP |
| β-actin | Proteintech Group | 66009-1-AP | 1:2000 for WB |
| STAT3 | Proteintech Group | 10253-2-AP | 1:1000 for WB, 1:50 for ChIP and IP |
| p-STAT3 | Affinity | YP0251 | 1:1000 for WB, 1:50 for IP, |
|  |  |  | 1:200 for IHC |
| H3 | Cell Signaling Technology | 17168-1-AP | 1:1000 for WB |
| H3K4ac | Immunoway | YK0192 | 1:1000 for WB, 1:200 for IHC |
| H3K4ac | Thermo Fisher | 712905 | 1:50 for ChIP |
| PKM1 | Cell Signaling Technology | #7067 | 1:1000 for WB |
| PKM1 | Proteintech Group | 15821-1-AP | 1:50 for RIP |
| SOX9 | Proteintech Group | 67439-1-Ig | 1:1000 for WB, 1:200 for IHC and mIHC |
| EP300 | Cell Signaling Technology | #86377S | 1:1000 for WB, 1:50 for ChIP |
| pan-Phospho | Thermo Fisher | 61-8300 | 1:50 for IP |
| HDAC3 | Abclonal | A19537 | 1:50 for ChIP |
| FITC Anti-Human CD44 | Elabscience | E-AB-F1215C | 1:100 for flow cytometry |
| APC Anti-Human CD117 | Elabscience | E-AB-F1150E | 1:100 for flow cytometry |

**Supplementary Table S3. Sequences of primers used for qRT-PCR.**

| **Target** |  | **Sequence** |
| --- | --- | --- |
| Linc00942 | Forward (5'-3') | TTGGAAACTTGCTGGGTGGT |
|  | Reverse (5'-3') | CTCAGAGGAGCGGAGGAATC |
| β-actin | Forward (5'-3') | AATCGTGCGTGACATTAAGGAG |
|  | Reverse (5'-3') | ACTGTGTTGGCGTACAGGTCTT |
| Linc00942 ΔP1 | Forward (5'-3') | CAGACGAAAGGAGTTCCCATAC |
|  | Reverse (5'-3') | GCAGACAAGATCTCACCTACAA |
| Linc00942 ΔP3 | Forword (5'-3') | AGCTGGCAAGACCTCTAAGC |
|  | Reverse (5'-3') | CTTTAGGCGTGAAGGAGGCT |
| SOX9 | Forword (5'-3') | GAGGAAGTCGGTGAAGAACG |
|  | Reverse (5'-3') | GATGCTGGAGGATGACTGC |
| SOX9 Promoter | Forward (5'-3') | GAAGGGAGGAGGGGTATTGC |
|  | Reverse (5'-3') | GATCACTCGCGTCTTGCTC |

**Supplementary Table S4. Sequences of primers used for RACE.**

| **Gene-specific primers** | **Sequence** |
| --- | --- |
| h-LINC00942-5RACE-F (93) | 5'-CAAGGAATCAGCTCTCCATTTCAGG-3' |
| h-LINC00942-5RACE-R1 (285) | 5'-GCAGACACCACCCAGCAAGTTTC-3' |
| h-LINC00942-3RACE-F1 (1851) | 5'-AGCAATTCTCCTGCCTCAGCCT-3' |
| h-LINC00942-5RACE-R2 (335) | 5'-GCAGTAGATTCTGAGCGTTTGCC-3' |
| 3RACE-F2 (1899) | 5'-CACCACCACGCCTGGCTAATTT-3' |
| 3RACE-R (2135) | 5'-CAGACCTTCCTCGGTTGCAGAGA-3' |

**Supplementary Table S5. Sequences of sgRNA for knockout of *Linc00942***

| sgRNA | Sequence |
| --- | --- |
| sgRNA1 | GAACAAGGACTTCCGTGCGA |
| sgRNA2 | AAGAATTCACGCTCGAAAGA |
| sgRNA3 | AATTGCCATTTGGGCTCCGA |
| sgRNA4 | CTGATCCCAGTCGCTACAAG |

**Supplementary Table S6. Sequences of shRNAs and siRNAs.**

| **Gene** | **Sequence** |
| --- | --- |
| SOX9 (sh-SOX9) | 5'- TATACAGAAATTGAAGGATGC -3' |
| lnc-ERC1-1:5 | Sense (5’-3’) UUUUGAGAUGGAGUUUCGCUC UUUUUUUUUUUUUGAGAUGGA Anti-sense (5’-3’) GCGAAACUCCAUCUCAAAAAA CAUCUCAAAAAAAAAAAAAAA |
| lnc-RP11-15K19.2.1-3:6 | Sense (5’-3’) ACAAAAUCAGUGAUAUACCAU AUGACAAUGGAUGUUAUUCUU Anti-sense (5’-3’) GGUAUAUCACUGAUUUUGUAG GAAUAACAUCCAUUGUCAUAC |
| lnc-SCG3-3:3 | Sense (5’-3’) UAAAAAGUCACCAAGUAACUU UCCUUUUUCUCCUUUUGGGCC Anti-sense (5’-3’) GUUACUUGGUGACUUUUUAUG CCCAAAAGGAGAAAAAGGAGC |
| Linc00942 | Sense (5’-3’) AAUUUUUAACACCUAAUGCUA UGUUCAAGGCCCACAAUCCAG Anti-sense (5’-3’) GCAUUAGGUGUUAAAAAUUAU GGAUUGUGGGCCUUGAACAUG |
| NR_036444 | Sense (5’-3’) ACAUUGUCUUGAAAUUUGGGU AUUUCACUUGGUAUCUUCGGA Anti-sense (5’-3’) CCAAAUUUCAAGACAAUGUUG CGAAGAUACCAAGUGAAAUAC |
| ENST00000602425 | Sense (5’-3’) AAUGUGAUAACUUAUCAGGUG UAAAAUGGGGUAGUAACAGCA  Anti-sense (5’-3’) CCUGAUAAGUUAUCACAUUGG CUGUUACUACCCCAUUUUACA |
| NONHSAT025399 | Sense (5’-3’) UGUUCAAGGCCCACAAUCCAG AUCAAAUGAGCCCUUAAGCAG Anti-sense (5’-3’) GGAUUGUGGGCCUUGAACAUG GCUUAAGGGCUCAUUUGAUGU |
| ENST00000549807 | Sense (5’-3’) UGACUUACAAUGAAAUCAGUA AAGGUAUUUUUUAGAUGUGAC Anti-sense (5’-3’) CUGAUUUCAUUGUAAGUCACA CACAUCUAAAAAAUACCUUCA |
| lnc-HIST1H2BI-1:1 | Sense (5’-3’) ACAAGUAUUUAAAUACUUCAC UAGAUUAUAAGAGUUUAGCUC Anti-sense(5’-3’) GAAGUAUUUAAAUACUUGUUU GCUAAACUCUUAUAAUCUAUA |
| lnc-TALC | Sense (5’-3’) AUAAUGACAUGUUACCAAGAG UUCAAAUGGCAAUCUUCAGAC Anti-sense (5’-3’) CUUGGUAACAUGUCAUUAUAA CUGAAGAUUGCCAUUUGAAAU |

**Supplementary Table S7. Details of vectors used in the present study.**

| Target | Manufacturer | Vector |
| --- | --- | --- |
| Linc00942 OE | Genechhem | Ubi-MCS-SV40-puromycin |
| LINC00942 KO | Genechhem | MCS-EF1a-Cas9-FLAG-P2A-puro |
| Linc00942 MUT1 | Genechhem | CMV-MCS-SV40-Neo |
| Linc00942 MUT2 | Genechhem | CMV-MCS-SV40-Neo |
| shSOX9 | Genechhem | hU6-MCS-SV40-Neo |
| Luciferase-Puro | Genechhem | Ubi-MCS-firefly_Luci-IRES-Puro |
| Luciferase-Neo | Genechhem | Ubi-MCS-firefly_Luci-SV40-Neo |

**Supplementary Table S8. Probe sequence for Linc00942 in FISH.**

| **Target** | **Probe sequence** |
| --- | --- |
| Linc00942-(1)  Linc00942-(2)  Linc00942-(3) | 5'- ACTTCAACATTGTTGTTTCCCTCTGAGGCTTGCAA-3  5'- GTTCCTGCTTCTGCTCTCCTCACACTGTTTCCTTA-3  5'- CATCTCCAGAAGGCTTGGAATTGATGACGTTTCCG-3 |

**Supplementary Table S9. ChIRP probes for Linc00942.**

| **Primers** | **Sequence** |
| --- | --- |
| Linc00942-P1 | 5'- CTTTGCCCTGAAATGGAGAG-3' |
| Linc00942-P2 | 5'- AGCAAGTTTCCAAGCAGGAG-3' |
| Linc00942-P3 | 5'- CTCCTCTGCAGAACTAACAC-3' |
| Linc00942-P4 | 5'- GAGGAATCTTTGGGAAGACT-3' |
| Linc00942-P5 | 5'- GCCCGTGTGTGTGAACAAAG-3' |
| Linc00942-P6 | 5'- TGCTCTTAACACCTTCTTTG-3' |
| Linc00942-P7 | 5'- CTCGATTCCTTCTGGATTAG-3' |
| Linc00942-P8 | 5'- TGTGAGGAGAGCAGAAGCAG-3' |
| Linc00942-P9 | 5'- TCTTGCTAGTCTAGCAAGTG-3' |
| Linc00942-P10 | 5'- TCTTGTGTTTGGAGGAGGAA-3' |
| Linc00942-P11 | 5'- TGTCTTCATTCTGATTCCTG-3' |
| Linc00942-P12 | 5'- GGCTGTTTGCAGTGTGAAAG-3' |
| Linc00942-P13 | 5'- CAAGCCTTCTGGAGATGAAC-3' |
| Linc00942-P14 | 5'- CAAGCTTATGTCGGATCGTG-3' |
| Linc00942-P15 | 5'- GAGGAAATCAGAGACGCCAG-3' |
| Linc00942-P16 | 5'- ATTTGGCCACTGAAGTCTAG-3' |
| Linc00942-P17 | 5'- TACAGGAAAGACTGGCTGGG-3' |
| Linc00942-P18 | 5'- TGAGATTATCCTGGGGAGAT-3' |
| Linc00942-P19 | 5'- TTAGGTGAAGTCTCTCAGGG-3' |
